# Algorithms for the adaptive assessment of procedural knowledge and skills

**DOI:** 10.3758/s13428-022-01998-y

**Published:** 2022-12-16

**Authors:** Andrea Brancaccio, Debora de Chiusole, Luca Stefanutti

**Affiliations:** Department of Philosophy, Sociology, Pedagogy, and Applied Psychology, University of Padua, Via Venezia, 14, 35131 Padova, Italy

**Keywords:** Procedural knowledge space theory, Problem space, Markov models, Adaptive assessment, Tower of London test

## Abstract

Procedural knowledge space theory (PKST) was recently proposed by Stefanutti (*British Journal of Mathematical and Statistical Psychology, 72*(2) 185–218, 2019) for the assessment of human problem-solving skills. In PKST, the problem space formally represents how a family of problems can be solved and the knowledge space represents the skills required for solving those problems. The Markov solution process model (MSPM) by Stefanutti et al. (*Journal of Mathematical Psychology, 103*, 102552, 2021) provides a probabilistic framework for modeling the solution process of a task, via PKST. In this article, three adaptive procedures for the assessment of problem-solving skills are proposed that are based on the MSPM. Beside execution correctness, they also consider the sequence of moves observed in the solution of a problem with the aim of increasing efficiency and accuracy of assessments. The three procedures differ from one another in the assumption underlying the solution process, named pre-planning, interim-planning, and mixed-planning. In two simulation studies, the three adaptive procedures have been compared to one another and to the continuous Markov procedure (CMP) by Doignon and Falmagne (1988a). The last one accounts for dichotomous correct/wrong answers only. Results show that all the MSP-based adaptive procedures outperform the CMP in both accuracy and efficiency. These results have been obtained in the framework of the Tower of London test but the procedures can also be applied to all psychological and neuropsychological tests that have a problem space. Thus, the adaptive procedures presented in this paper pave the way to the adaptive assessment in the area of neuropsychological tests.

**Supplementary Information:**

The online version contains supplementary material available at 10.3758/s13428-022-01998-y.

## Introduction

In this article, a novel procedure for the adaptive assessment of human problem-solving is presented, which is suitable for performing the assessment with certain cognitive or neuropsychological tests like, for instance, the Tower of London (ToL) test. The theory on which the procedure is based is named *procedural knowledge space theory* (Stefanutti, 2019). It is a specialization of the knowledge structures theory (KST; Doignon & Falmagne, 1985; 1999; Falmagne & Doignon, 2011) to the formal modeling and the assessment of human problem-solving. In particular, the procedures presented in this article are on well-structured, finite problems and problem spaces.

Problem-solving is a prominent activity of humans. As such, it arises in many areas of human life. Given its importance, there is an abundance of literature having problem-solving as the main or a secondary research topic. For instance, (Jonassen, 2000) proposed a typology of 11 types of problems considered in problem-solving studies, from well-structured logical problems to ill-structured dilemmas. Moreover, (Funke, 2013) presented an extensive bibliography of 263 studies related to human problem-solving for further references. Those studies include several fields, such as education, neuroscience, and artificial intelligence.

Formal and probabilistic models of problem-solving have been developed within KST (Falmagne, Albert, Doble, Eppstein, & Hu, 2013) and also in the area of the so-called cognitive diagnostic models (CDM; Bolt, 2007; de la Torre, 2009; DiBello & Stout, 2007; Tatsuoka, 1990). Such theories are based on a problem-to-skills relationship which provides the fundamental skeleton of the developed models.

PKST is built upon the notion of a “problem space” (Newell & Simon, 1972), and it is applicable to all and only those problem situations for which a problem space exists and can be given. As such, PKST is at the meeting point between the theory of problem spaces (Newell & Simon, 1972) and that of knowledge spaces (Doignon & Falmagne, 1985).

In the original definition by Newell and Simon (1972), a “problem space” is the internal representation that a problem solver makes of a given task environment. Then, problem-solving consists of exploring this internal representation, in search of a solution. Very often, in the literature (see, e.g., Langley, Magnani, Schunn, & Thagard, 2005; Zhang & Norman, 1994), the term “problem space” also refers to a conceptual structure that can be objectively constructed and displayed (e.g., by a computer program) by repeatedly applying a finite set of transformation rules, starting from the initial configuration of the problem. In this article, the term “problem space” refers to this objectively obtainable structure. A classical example of such a construction is offered by the problem space of the Tower of Hanoi, described by Newell and Simon (1972). Another example, which is extensively described and applied in this article, is the problem space of the Tower of London test, a rather well-known neuropsychological test of executive functions (Shallice, 1982).

In PKST, the problem space represents complete knowledge over the problem. It is all a perfect problem solver needs to know for successfully solving a given set of problems. Such an ideal representation is based on properties that need not be satisfied by the knowledge state of an imperfect problem solver (e.g., a human one). Indeed, at least two sources of “imperfect” answers can occur in practice. The former deals with a sort of intransitivity of the human cognitive capability, in the sense that being able of solving two distinctive sub-problems does not necessary mean being able to solve the problem that concatenates those two sub-problems. The latter deals with the incomplete knowledge over the problem domain. In this case, the knowledge state of a problem solver is a strict subset of the whole problem space (a *problem subspace*). PKST is about the knowledge states of both perfect and imperfect problem solvers, the collection of which is named the *procedural knowledge space*.

Both the problem space and the procedural knowledge space are deterministic models. As such, they cannot be empirically validated, for instance, by means of standard goodness-of-fit statistics. A probabilistic model that incorporates all the critical deterministic assumptions of PKST has been recently developed by Stefanutti et al., (2021). It is based on the notion of a Markov solution process (MSP), a stochastic process that represents the problem solution behavior of a problem solver.

The MSP model (henceforth MSPM) can be used for uncovering (inferring) the knowledge state of an individual, on the basis of the solution behavior observed in a given subset of problems of the problem space. In this article, a novel adaptive assessment procedure, based on the MSPM, is described. The procedure features many interesting aspects. In the first place, being an adaptive procedure, it minimizes the number of questions and, at the same time, it maximizes the information on the underlying state of knowledge. Problem spaces may be large, containing hundreds or even thousands of different problems and sub-problems. To give an example, the problem space of the ToL contains in the whole 1260 distinct problems, but the test by Shallice (1982) only uses 12 of them. What type of inference can be done from these fixed 12 problems to the remaining 1248, for every single individual, is not immediately obvious. The proposed procedure may be used for making inferences over the whole problem domain on the basis of a reasonably small subset of problems, which is tailored to the individual.

In the second place, existing adaptive assessment procedures in KST are not trivially applicable to response data that, going beyond the correct/incorrect response format, keep track of the whole trail of moves performed in intermediate steps of the problem solution process. The capability of exploiting this surplus of information, which arises naturally in problem-solving, is the most critical and important feature of the proposed procedure.

The third distinctive feature of the procedure is the assessment paradigm on which it is based. In a problem space, the order of difficulty of the problems could fail to be linear (i.e., from the easiest to the most difficult). There is a quite natural assumption for the problems in a problem space that provides a reason for this: If a person can solve a problem by following a specific solution path along the problem space, then, excluding random error, that person will be able to solve all the sub-problems that are encountered along that path. In general, this assumption induces an order of difficulty on the problems which is only partial. In PKST, this assumption is named the “sub-path assumption”. Therefore, PKST does not impose any strong measurement requirements to data. Items do not need to be all aligned along a unidimensional continuum, and there is no need to throw away items that do not conform to this requirement.

The manuscript is organized as follows. Backgrounds are given in “Background”, whereas the proposed adaptive assessment procedures are presented in “Adaptive assessment in a problem space”. In both “Background” and “Adaptive assessment in a problem space” the theoretical explanations are illustrated with practical examples. In “Simulation study” and “Simulation study based on real data”, three MSPM-based procedures were compared in two simulation studies. In “Simulation study”, a series of simulation studies were carried out with the aim of testing how different assumptions concerning human planning affect the capability of the procedures to predict the actual planning skills of an individual. In “Simulation study based on real data”, some simulations were run by using a pre-existing data set consisting of the responses of 154 participants to a subset of Tower of London problems. A general discussion concludes the article (“General discussion”).

## Background

Different theoretical frameworks contribute to the state of the art of the present research. A section for each of these topics follows.

### The Tower of London test

Throughout the article, the various concepts of PKST are illustrated with the help of the example of the Tower of London test (Shallice, 1982). In particular, “Simulation study” and “Simulation study based on real data” describe extensive applications of PKST to the ToL test. For these reasons, the ToL is briefly described here.

**Fig. 1:**
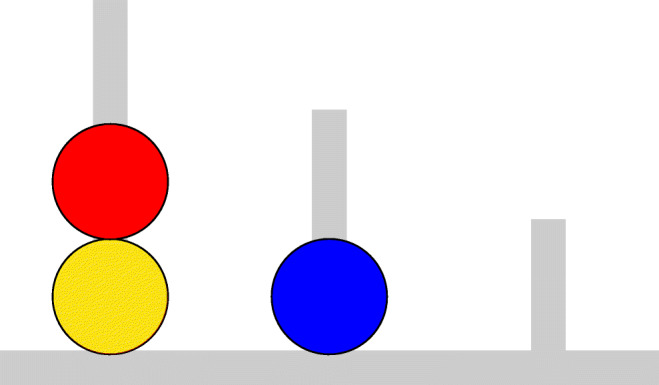
Problem state 31 of the Tower of London test

The ToL was developed by Shallice (1982) for assessing planning deficits in patients with lesions of the frontal lobe. Today, it is used for assessing planning ability in the clinical and non-clinical population (Berg and Byrd, 2002). The ToL consists in three equally spaced pegs with different heights, mounted on a wooden support. An example of the spatial configuration of the ToL is illustrated in Fig. 1.

**Fig. 2:**
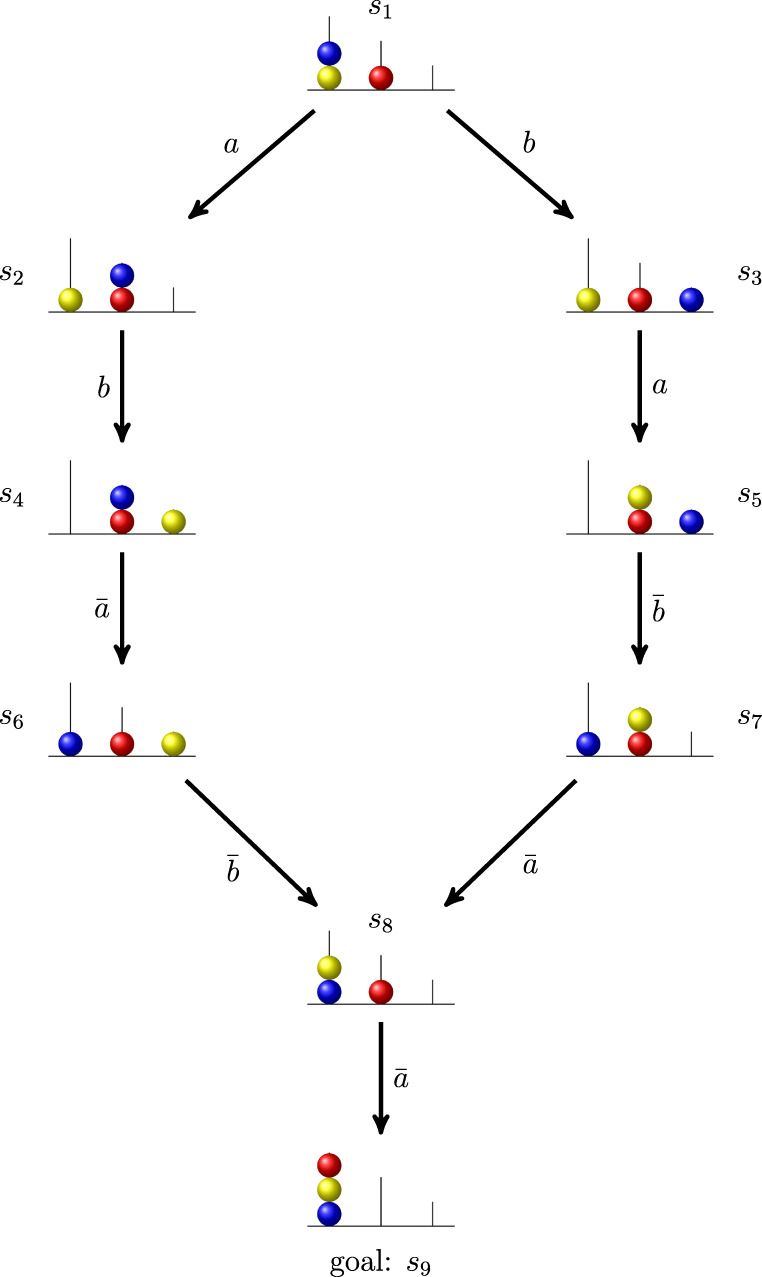
Portion of the problem space of the Tower of London test representing two solution paths of a five-moves problem

In total, there are 36 spatial configurations, each of which forms a different problem state. The three balls of different colors can be moved, one at the time, from one peg to another. Each problem consists of transforming a certain initial configuration, named *initial state*, into a final configuration, called *goal state*. For instance, in Fig. 2, where a portion of the ToL problem space is represented, the pair of problem states *s*_4_,*s*_9_ can be seen respectively, as the initial state and the goal state of a problem. The task is correctly performed if the goal state is obtained with a minimum number of moves. Thus, to avoid mistakes, the problem solver must plan the sequence of moves in advance.

In the original ToL test, developed by Shallice (1982), an indirect measure of the difficulty of a problem is obtained as the minimum number of moves necessary to solve it. However, recent studies (e.g., Berg, Byrd, McNamara, & Case, 2010; Kaller, Unterrainer, Rahm, & Halsband, 2004; Kaller, Rahm, Köstering, & Unterrainer, 2011; McKinlay et al., 2008; Newman & Pittman, 2007) found that other factors affect the difficulty of a problem. Some of them are the number of alternative solutions for the problem, the initial configuration of the balls on the pegs (named “start hierarchy”), and the final configuration (named “goal hierarchy”). As it will be seen, the approach proposed in this article goes much beyond the notion of minimum number of moves.

As already mentioned, the problem space of the ToL consists in 6 × 6 = 36 different problem states obtained as the Cartesian product of the six different permutations of the three colors times the six spatial arrangements of the balls in the pegs. In the sequel, every single problem state in the ToL problem space is uniquely referred to by using a pair *ab* of numbers, where *a* stands for one of the six spatial arrangements whereas *b* stands to one of the six color permutations. The reader is referred to Stefanutti et al., (2021) for the complete list of problem states codings.

### Knowledge space theory

The theory of knowledge spaces (Doignon and Falmagne, 1985; 1999; Falmagne & Doignon, 2011) is a mathematical approach to a non-numerical assessment of knowledge. In KST, the *domain of knowledge* is the nonempty set *Q* of all the problems in a given field of knowledge (e.g., mathematics, chemistry, statistics, etc.). The *knowledge state* of a student is the set $K \subseteq Q$ of all the problems that she is able to solve. The *knowledge structure* is the collection $\mathcal {K}$ of all the knowledge states. By definition, $\mathcal {K}$ always contains both the empty set and *Q*. A knowledge structure is named *knowledge space* if, for any subfamily $\mathcal {F} \subseteq \mathcal {K}$, the union of the subsets in $\mathcal {F}$ is still in $\mathcal {K}$.

KST was initially developed as a behavioral theory, in the sense that it provided no assumptions or descriptions of cognitive processes, skills, or resources behind the solution of a problem. Later, the theory was extended to the assessment of skills (Doignon, 1994; Düntsch & Gediga, 1995; Falmagne, Koppen, Villano, Doignon, & Johanessen, 1990; Gediga & Düntsch, 2002; Stefanutti & de Chiusole, 2017; Ünlü et al., 2013; Heller, Stefanutti, Anselmi, & Robusto, 2015; Korossy, 1997; Korossy, 1999). Such extension is known as *competence-based knowledge space theory* (CbKST; Heller, Ünlü, & Albert, 2013; Heller, Augustin, Hockemeyer, Stefanutti, & Albert, 2013; Stefanutti & Albert, 2003). Given a set π of skills, the *competence state* is the set $C \subseteq {\Pi }$ of skills mastered by an individual. The collection $\mathcal {C}$ of all the competence states is the *competence structure*. The problems and the skills are related by a *skill map* (Doignon, 1994), which is a triple (*Q*,π,*τ*) where $\tau : Q \rightarrow 2^{\Pi }$ is a function assigning to each problem in *Q* a non-empty subset of skills in π.

### Procedural knowledge space theory

*Procedural knowledge space theory* (Stefanutti & Albert, 2003; Stefanutti, 2019) generalizes the application of KST and CbKST to the area of human problem-solving and procedural knowledge.

Let Ω be a set of operations. For example, in the ToL there are six operations each of which move a ball from one peg to another, and in particular, naming the three pegs as left, center and right, one has: (*a*) left to center; (*b*) center to right; (*c*) left to right;$(\bar a)$ center to left; $(\bar b)$ right to center; $(\bar c)$ right to left. Therefore, in the ToL, the set of operations is ${\Omega }_{\text {ToL}} = \{a,b,c,\bar a,\bar b,\bar c\}$.

A sequence of operations in Ω is denoted as *ω*_1_*ω*_2_⋯*ω*_*n*_. Given two sequences of operations in Ω, *α* = *ω*_1_*ω*_2_⋯*ω*_*m*_, *β* = *ω*_*m*+ 1_*ω*_*m*+ 2_⋯*ω*_*n*_ their concatenation is the sequence *αβ* = *ω*_1_*ω*_2_⋯*ω*_*m*_*ω*_*m*+ 1_*ω*_*m*+ 2_⋯*ω*_*n*_. The collection of all the sequences of operations of arbitrary finite length, including the empty sequence *𝜖* is

$${\Omega}^{*} = \bigcup\limits_{n \in \mathbb{Z}^{+}} {\Omega}^{n},$$

where $\mathbb {Z}^{+}$ is the set of the non-negative integer numbers.

A *problem space* is formally defined as a triple **P** = (*S*,Ω,⋅), in which *S* is a nonempty set of problem states, Ω is a non-empty set of operations, and $\cdot : S \times {\Omega }^{*}\rightarrow S$ is an operator that satisfies the following properties:

1. *s* ⋅ *𝜖* = *s*,
2. (*s* ⋅ *σ*)*π* = *s* ⋅ *σπ*,

where *s* ∈ *S* and *σ*,*π* ∈Ω^∗^. The operator ⋅ is called *operation application*.

Figure 2 shows the directed graph of a portion of the problem space of the ToL test. Each vertex in the graph corresponds to a problem state in the set *S*_ToL_. This last contains nine of the 36 problem states of the ToL. The directed edges of the graph are labeled by the moves in Ω_ToL_.

A directed edge in the figure links a problem state *s* to another problem state *t* if there is a move in Ω_ToL_ transforming *s* into *t*.

A problem in a problem space is any pair (*s*,*t*) of problem states, with *s*≠*t*, such that *s* ⋅ *π* = *t* for some sequence *π* of operations in Ω^∗^. Stated differently, a pair (*s*,*t*) is a problem if, by applying the sequence *π* to the problem state *s*, the problem state *t* is obtained. State *s* is named the *initial state* of the problem, whereas *t* is the *goal state*.

In the running example of ToL, the pair (*s*_2_,*s*_9_) of problem states in Fig. 2 is a problem because the sequence of operations $b \bar {a} \bar {b} \bar {a}$ transforms the initial problem state *s*_2_ into the goal problem state *s*_9_.

The set of all the problems in **P** is thus

$$ Q = \{(s,t) \in S \times S: s \neq t ~\text{and}~ s \cdot \pi \in t ~\text{for some}~ \pi \in {\Omega}^{*}\}. $$

It is worth noticing that the set *Q* obtained in this way is nothing else than what in KST is named the domain of knowledge. Any pair *sπ* (without the dot in between) is called a *solution path*. The solution path *sπ* *solves* problem (*s*,*t*) ∈ *Q* if *s* ⋅ *π* = *t*. The set of all the solution paths turns out to be π = *S* × (Ω^∗^∖{*𝜖*}).

In the subsequent example, only a part of the whole set of problems for the problem space in Fig. 2 is considered, namely *Q*_ToL_ = {(*s*_1_,*s*_9_),(*s*_3_,*s*_9_),(*s*_4_,*s*_9_),(*s*_7_,*s*_9_), (*s*_8_,*s*_9_)}. Since all the problems in *Q*_ToL_ have form (*s*_*i*_,*s*_9_), for lightening the notation, each of them is just represented by the initial state *s*_*i*_. To solve a problem, one needs to know at least one of the solution paths of that problem. For instance, problem *s*_1_ has two possible solution paths, namely $s_{1} a b \bar {a} \bar {b} \bar {a}$ and $s_{1} b a \bar {b} \bar {a} \bar {a}$. It is left to the reader to check that the set of all solution paths that solve any one of the problems in *Q*_ToL_ is

$$ {\Pi}_{\text{ToL}} = \{s_8\bar{a},s_7\bar{a}\bar{a}, s_4\bar{a}\bar{b}\bar{a}, s_3a\bar{b}\bar{a}\bar{a}, s_1ab\bar{a}\bar{b}\bar{a}, s_1ba\bar{b}\bar{a}\bar{a}\}. $$

Solution paths are partially ordered. Precisely, a solution path *sπ* is a *subpath* of another solution path *tσ* (denoted by $s\pi \sqsubseteq t\sigma $) if there are *α*,*β* ∈Ω^∗^ such that *σ* = *απβ* and *t* ⋅ *α* = *s*. For instance, in Fig. 2, consider the two solution paths $s_{4} \bar {a}\bar {b}\bar {a}$ and $s_{1} ab\bar {a}\bar {b}\bar {a}$. It is easily seen that the former is a subpath of the latter. In fact, by setting *α* = *ab* and *β* = *𝜖*, it holds that $ ab\bar {a}\bar {b}\bar {a}=\alpha \bar {a}\bar {b}\bar {a}\beta $, and *s*_1_ ⋅ *α* = *s*_4_. The cognitive interpretation of the subpath relation is that if an individual knows a solution path, then she will also know all of its solution subpaths.

A solutions path can be seen as a kind of “procedural skill” required for solving a problem. Therefore, the collection of all the solution paths solving a certain problem (*s*,*t*) ∈ *Q* is denoted *τ*(*s*,*t*), where *τ* : *Q* → 2^π^ is a mapping having *Q* as the domain and the powerset of π as the codomain. Using the Cb-KST notation, the triple (*Q*,π,*τ*) is named the *skill map* derived from the problem space **P**. In this example, for the sake of simplicity, the mapping *τ*_ToL_ for the subset π_ToL_ is constructed instead of deriving the mapping *τ* for the whole set π of solution paths. The mapping *τ*_ToL_ is defined as follows:

$$ \begin{array}{@{}rcl@{}} \tau_{\text{ToL}}(s_{1},s_{9}) &=& \{s_{1}ab\bar{a}\bar{b}\bar{a}, s_{1}ba\bar{b}\bar{a}\bar{a}\}, \\ \tau_{\text{ToL}}(s_{3},s_{9}) &=& \{s_{3}a\bar{b}\bar{a}\bar{a}\}, \\ \tau_{\text{ToL}}(s_{4},s_{9}) &=& \{s_{4}\bar{a}\bar{b}\bar{a}\}, \\ \tau_{\text{ToL}}(s_{7},s_{9}) &=& \{s_{7}\bar{a}\bar{a}\}, \\ \tau_{\text{ToL}}(s_{8},s_{9}) &=& \{s_{8}\bar{a}\}. \end{array} $$

A subset $C \subseteq {\Pi }$ is said to *respect path inclusion* if the condition

$$ s\pi \sqsubseteq t\sigma, t\sigma \in C \implies s\pi \in C $$

is respected for all *sπ*,*tσ* ∈π. A subset of solution paths respecting path inclusion is named a *competence state* of the problem space **P**. The collection $\mathcal {C}$ of all the competence states is the *competence space*. In the running example of ToL, the collection $\mathcal {C}_{\text {ToL}}$ of all the solution paths in π_ToL_ that respect the path inclusion is

$$ \begin{array}{@{}rcl@{}} \mathcal{C}_{\text{ToL}}= &&\{\emptyset,\{s_{8}\bar{a}\},\{s_{8}\bar{a},s_{7}\bar{a}\bar{a}\},\{s_{8}\bar{a},s_{4}\bar{a}\bar{b}\bar{a}\},\\ &&\{s_{8}\bar{a},s_{7}\bar{a},s_{4}\bar{a}\bar{b}\bar{a}\}, \{s_{8}\bar{a},s_{7}\bar{a}\bar{a},s_{3}a\bar{b}\bar{a}\bar{a}\}, \\ &&\{s_{8}\bar{a},s_{4}\bar{a}\bar{b}\bar{a},s_{1}ab\bar{a}\bar{b}\bar{a}\}, \{s_{8}\bar{a},s_{7}\bar{a}\bar{a},s_{4}\bar{a}\bar{b} \bar{a},s_{1}ba\bar{b}\bar{a}\bar{a}\},\\ &&\{s_{8}\bar{a},s_{7}\bar{a}\bar{a},s_{4}\bar{a}\bar{b} \bar{a},s_{3}a\bar{b}\bar{a}\bar{a}\}, \\ &&\{s_{8}\bar{a},s_{7}\bar{a}\bar{a},s_{3}a\bar{b}\bar{a}\bar{a},s_{1}ab\bar{a}\bar{b}\bar{a}\},\\ &&\{s_{8}\bar{a},s_{7}\bar{a}\bar{a}, s_{4}\bar{a}\bar{b}\bar{a},s_{3}a\bar{b}\bar{a}\bar{a},s_{1}ab\bar{a}\bar{b}\bar{a}\}\\ &&\{s_{8}\bar{a},s_{7}\bar{a}\bar{a}, s_{4}\bar{a}\bar{b}\bar{a},s_{3}a\bar{b}\bar{a}\bar{a},s_{1}ba\bar{b}\bar{a}\bar{a}\},\\ &&\{s_{8}\bar{a},s_{7}\bar{a}\bar{a}, s_{4}\bar{a}\bar{b}\bar{a},s_{3}a\bar{b}\bar{a}\bar{a},s_{1}ba\bar{b}\bar{a}\bar{a},s_{1}ab\bar{a}\bar{b}\bar{a}\} \}. \end{array} $$

The set of all the problems in *Q* that can be solved by an individual whose competence state is $C \in \mathcal {C}$ is given by the *problem function*, which is defined as

$$ p(C) = \{(s,t) \in Q: \tau(s,t) \cap C \ne \emptyset\}. $$

Thus, *p*(*C*) contains all and only those problems (*s*,*t*) that can be solved by one or more solution paths, among those contained in *C*. Each such problem satisfies the condition *τ*(*s*,*t*) ∩ *C*≠*∅*. The set *p*(*C*) is named the *knowledge state* delineated by the competence state *C*. The collection $\mathcal {K}=\{p(C): C \in \mathcal {C}\}$ of all the knowledge states is the *knowledge space* derived from the problem space **P**.

For instance, in the running example of the ToL, the knowledge state delineated by the competence state $\{s_{8}\bar {a},s_{7}\bar {a}\bar {a},s_{4}\bar {a}\bar {b}\bar {a}\}$ is

$$ p(\{s_{8}\bar{a},s_{7}\bar{a}\bar{a},s_{4}\bar{a}\bar{b}\bar{a}\})=\{s_{8},s_{7},s_{4}\}. $$

In the whole, if the problem function *p* is applied to each of the competence states, the following knowledge space is obtained:

$$ \begin{array}{@{}rcl@{}} \mathcal{K}_{\text{ToL}} &=& \{\emptyset,\{s_{8}\},\{s_{4},s_{8}\},\{s_{7},s_{8}\},\{s_{4},s_{7},s_{8}\}, \{s_{1},s_{4},s_{8}\},\\ &&\{s_{3},s_{7},s_{8}\},\{s_{1},s_{4},s_{7},s_{8}\}, \\ &&\{s_{3},s_{4},s_{7},s_{8}\}, \{s_{1},s_{3},s_{7},s_{8}\}, \{s_{1},s_{3},s_{4},s_{7},s_{8}\}\}. \end{array} $$

### The continuous Markov procedure

Adaptive assessment is one of the most important applications in knowledge space theory. The aim of an adaptive assessment is to uncover the individual knowledge state with a minimal number of questions. Some examples of this procedure are present in fields such as education (see, e.g., ALEKS, www.aleks.com, and Stat-Knowlab, de Chiusole, Stefanutti, Anselmi, & Robusto, 2020), and psychological assessment (Donadello et al., 2017; Granziol et al., 2020). In KST, the standard procedure used for implementing the adaptive assessment is the continuous Markov procedure by Falmagne and Doignon (1988). It is an iterative procedure which uses a likelihood distribution ${\mathscr{L}}_{m}:\mathcal {K} \to \mathbb {R}$ with the collection $\mathcal {K}$ as the domain and the $\mathbb {R}$ as codomain. The likelihood distribution is updated at each step *m* of the procedure on the basis of the incoming information. Unless prior information is available, the initial likelihood distribution ${\mathscr{L}}_{0}$ is the uniform one. At each step *m*, the procedure: (i) selects a new problem for the student; (ii) updates the likelihood distribution on the knowledge states depending on the student’s response; (iii) establishes if enough information has been collected and in that case, terminates. Different rules were proposed by Falmagne and Doignon (1988) and Doignon and Falmagne (1999) for each of these three phases. The rules that are relevant with respect to this article are described below.

The questioning rule selects a problem *q* ∈ *Q* in order to minimize the total number of questions to be administered before the assessment terminates. One such rule is the so-called *half-split* (Falmagne & Doignon, 2011), in which any one of the problems *q* ∈ *Q* is selected among those that minimize the following quantity:

1 $$ \mathbf{Q}_{m}=\underset{q \in Q}{\arg\min} \lvert 2 \cdot \mathcal{L}_{m}(\mathcal{\mathcal{K}}_{q})-1 \rvert , $$

where $\mathcal {K}_{q} =\{ K \in \mathcal {K} : q \in K\}$ and ${\mathscr{L}}_{m}(\mathcal {K}_{q})= {\sum }_{K \in \mathcal {K}_{q}}$ ${\mathscr{L}}_{m}(K) $.

The updating rule updates the likelihood ${\mathscr{L}}_{m}$ on the basis of the answer collected at the step *m* of the procedure. Whenever the student’s response is correct (incorrect), the likelihood ${\mathscr{L}}_{m}(K)$ of all $K \in \mathcal {K}$ such that *q* ∈ *K* increases (decreases), whereas the likelihood ${\mathscr{L}}_{m}(K^{\prime })$ for all $K^{\prime } \in \mathcal {K}$ such that $q \notin K^{\prime }$ decreases (increases). The likelihood function is updated at each step *m* + 1 of the assessment procedure by following a *Bayesian* updating rule:

2 $$ \mathcal{L}_{m+1}(K)=\frac{P(r_{q}|K) \mathcal{L}_{m}(K)}{{\sum}_{K^{\prime} \in \mathcal{K}}P(r_{q}|K) \mathcal{L}_{m}(K^{\prime})}, $$

where the parameter *P*(*r*_*q*_|*K*) represents the conditional probability of the observed response *r*_*q*_ for item *q* given the knowledge state *K*. In the procedure by Falmagne and Doignon (1988), two types of probabilities are defined for each item *q*—a careless error probability *β*_*q*_ and a lucky guess probability *η*_*q*_. Then, the *P*(*r*_*q*_|*K*) parameter undergoes the following constraints:

3 $$  P(r_{q}|K) = \begin{cases} \beta_{q} & \text{if } r_{q}=0 \text{ and } q \in K;\\ 1-\eta_{q} & \text{if } r_{q}=0 \text{ and } q \notin K;\\ 1-\beta_{q} & \text{if } r_{q}=1 \text{ and } q \in K;\\ \eta_{q} & \text{if } r_{q}=1 \text{ and } q \notin K.\\ \end{cases} $$

Equation 3 is known as the *response rule*.

The procedure continues to select questions and to update the likelihood until a termination criterion is reached. The most used termination criterion consists of fixing a threshold *p* that has to be reached by the maximum of the likelihood distribution ${\mathscr{L}}_{m}$. The minimum value of such a threshold is .50 because this is a sufficient condition for have a unimodal likelihood distribution. In general, the accuracy of the assessment improves when *p* approaches 1, and this occurs at the expense of efficiency. In fact, the larger *p*, the larger the expected number of questions that have to be administered.

An alternative representation of this updating rule, also known as the *multiplicative rule*, is defined as follows:

4 $$ \mathcal{L}_{m}(K)=\frac{\zeta_{q,r_{q}}^{K} \mathcal{L}_{m}(K)}{{\sum}_{K^{\prime} \in \mathcal{K}}\zeta_{q,r_{q}}^{K^{\prime}} \mathcal{L}_{m}(K^{\prime})}, $$

where the parameters $\zeta ^{K}_{q,r_{q}}$ depends on the knowledge state $K \in \mathcal {K}$, the problem *q* ∈ *Q*, and the observed response *r*_*q*_. In particular, $\zeta ^{K}_{q,r_{q}}$ is defined as follows:

5 $$ \zeta_{q,r_{q}}^{K} = \begin{cases} \zeta_{q,1} & \text{if } r_{q}= 1 \text{ and } q \in K;\\ 1 & \text{if } r_{q}= 1 \text{ and } q \notin K;\\ 1 & \text{if } r_{q}= 0 \text{ and } q \in K;\\ \zeta_{q,0} & \text{if } r_{q}= 0 \text{ and } q \notin K.\\ \end{cases} $$

where *ζ*_*q*,0_ and *ζ*_*q*,1_ > 1 are real parameters of the assessment procedure.

Moreover, (Falmagne & Doignon, 1988) have shown that the Bayesian updating rule is equivalent to the multiplicative rule under the following equalities, for each *q* ∈ *Q*:

6 $$ \zeta_{q,1}= \frac{1-\beta_{q}}{\eta_{q}} \text{ and } \zeta_{q,0}= \frac{1-\eta_{q}}{\beta_{q}}. $$

A latent knowledge state $K_{0} \in \mathcal {K}$ is said to be *uncoverable* by the stochastic assessment procedure presented above if ${\mathscr{L}}_{m}(K_{0})$ approaches 1 almost surely.

Several theoretical results were obtained for the multiplicative updating rule. One of them is important here because it will be used in Section “Adaptive assessment in a problem space”.

#### **Proposition 1**

A latent knowledge state is uncoverable by a stochastic assessment procedure with an updating rule which is multiplicative and a questioning rule which is half-split.

### The Markov solution process model

A Markov model of the solution process of a problem-solving task was proposed in Stefanutti et al., (2021). The model provides a stochastic framework for the deterministic models described in Section “Procedural knowledge space theory”. It has been empirically validated for the case of the ToL test (Stefanutti et al., 2021), where it obtained a satisfactory goodness-of-fit.

A central notion for the application of the Markov model is that of a *goal space* where each step of the solution process of a problem is classified as “correct” or “incorrect”. A goal space is a problem space where there are two special problem states *f*,*g* ∈ *S* that are labeled the *failure* and *goal* states, respectively. Every problem in a goal space has the form (*s*,*g*), with *s* ∈ *S* ∖{*f*}. The formal definition of the goal space is as follows.

#### **Definition 1**

A problem space (*S*,Ω,⋅) is a goal space if distinct states *f*,*g* ∈ *S* exist such that:

(**GS1**): for all *ω* ∈Ω, *f* ⋅ *ω* = *f* and *g* ⋅ *ω* = *g*;
(**GS2**): for each *s* ∈ *S* ∖{*f*} there is a string *π* ∈Ω^∗^ such that *s* ⋅ *π* = *g*.

A goal space is denoted by the 5-tuple (*S*,*f*,*g*,Ω,⋅).

It follows from Condition (GS1) of Definition 1 that *f* and *g* are final states. In particular, whenever the solution process of a problem enters either *g* or *f*, the problem is marked as “correct ” or “incorrect”, respectively, and the solution process terminates. According to Condition (GS2), each problem state different from *f* has a solution path that terminates in *g*. The graph represented in Fig. 2 is an example of a goal space, where *s*_9_ is the goal state. The failure state is omitted in the figure, but it could be easily added as a state that can be reached by every non-goal state.

Let $(Q,\mathcal {K})$ be the knowledge space derived from the goal space (*S*,*f*,*g*,Ω,⋅). The behavior of a problem solver in knowledge state $K \in \mathcal {K}$, who is attempting to solve problem (*s*,*g*), is modeled as a random process $\mathbf {S}=\{\mathbf {S}_{n}:n\in \mathbb {Z}^{+}\}$ that satisfies the following Markov property:

7 $$ \begin{array}{@{}rcl@{}} P(\mathbf{S}_{n} &=& s_{n}|\mathbf{S}_{n-1} = s_{n-1}, \mathbf{S}_{n-2} = s_{n-2},\ldots,\mathbf{S}_{0} = s; s, K)\\ &=& P(\mathbf{S}_{n} = s_{n}|\mathbf{S}_{n-1} = s_{n-1};s,K). \end{array} $$

Property 7 says that, given the last visited problem state **S**_*n*− 1_, the knowledge state *K* of the problem solver, and the initial state *s*, the next problem state **S**_*n*_ is independent of the past history of the process. For the right-hand term of Eq. 7 the shortcut notation *P*(*s*_*j*_|*s*_*i*_,*s*,*K*) is used, which is named the *transition probability* from state *s*_*i*_ to state *s*_*j*_.

Even with problem spaces and related knowledge spaces of moderate size, the number of transition probabilities of this type could be huge. The *Markov solution process model* provides a reasonable assumption that allow to drastically reduce the number of free parameters of the model by introducing constraints on transition probabilities. Let *E* = {(*s*,*t*) ∈ *S*^2^ : *s* ⋅ *ω* = *t* for some *ω* ∈Ω} be the collection of all the *elementary problems* (i.e., problems each of which can be solved by a single operation). Then the assumption is:(MSP1) For every problem (*s*,*g*) ∈ *Q*, every pair (*i*,*j*) ∈ *E* and every knowledge state $K \in \mathcal {K}$,

8 $$ P(j|i,s,K) = \begin{cases} \beta_{ij} & \text{if $(s,g) \in K$,}\\ \eta_{ij} & \text{if $(s,g) \in Q \setminus K$,} \end{cases} $$

where *β*_*ij*_ and *η*_*ij*_ are free parameters of the model.

In the MSP1 assumption, given any pair (*i*,*j*) ∈ *E*, the value of the transition probability from *i* to *j* is either *β*_*ij*_ or *η*_*ij*_, depending on whether the problem (*s*,*g*) belongs to *K* or not. In particular, if *i* is a transient problem state, then the parameter *β*_*if*_ is the probability that a problem solver which knows at least one solution path for (*s*,*g*) made a careless error. Similarly, for *j*≠*f*, the parameter *η*_*ij*_ is the probability that a problem solver who does not know any solution path for (*s*,*g*) guesses a correct move from *i*. Further details of the MSPM are not presented here, since they are not needed in the sequel. For a complete exposition of the model, the reader is referred to Stefanutti et al., (2021).

## Adaptive assessment in a problem space

In many psychological tests (e.g., the Tower of London test, Tower of Hanoi, mental rotation task), the different tasks are accomplished by performing a sequence of observable moves. The CMP described in “The continuous Markov procedure” is based on dichotomous answers (i.e., correct or incorrect) and it has no mechanism for capitalizing on the information provided by the observable solution process. The following example shows the drawbacks of this limitations.

### *Example 1*

Consider the knowledge space $\mathcal {K}_{\text {ToL}}$ derived in the running example in “Procedural knowledge space theory”. Suppose that the state of a problem solver is {*s*_1_,*s*_3_,*s*_4_,*s*_7_,*s*_8_}, and that the CMP is applied for uncovering it. The beta parameters for the five problems are assumed to be $\beta _{s_{1}}=.004, \beta _{s_{3}}=.03, \beta _{s_{4}}=.02, \beta _{s_{7}}= .01,$ and $\beta _{s_{8}}= .007$, whereas the eta parameters are assumed to be $\eta _{s_{1}}=10^{-6}, \eta _{s_{3}}=5 \times 10^{-5}, \eta _{s_{4}}= 4 \times 10^{-5}, \eta _{s_{7}}= .007,$ and $\eta _{s_{8}}= .08$. At the beginning of the assessment (*m* = 0), all of the knowledge states $K \in \mathcal {K}_{\text {ToL}}$ have the same likelihood ${\mathscr{L}}_{0}(K)= 1/ |\mathcal {K}_{\text {ToL}}|$ (see the second column of Table 1).

**Table 1:** Values of the likelihood distribution ${\mathscr{L}}_{m}$ at each step *m* of the assessment procedure

*K*	$ {\mathscr{L}}_{0}(K)$	$ {\mathscr{L}}_{1}(K)$	$ {\mathscr{L}}_{2}(K)$	$ {\mathscr{L}}_{3}(K)$
{*∅*}	.09	0	0	0
{*s*_8_}	.09	0	0	0
{*s*_7_,*s*_8_}	.09	0	0	0
{*s*_4_,*s*_8_}	.09	.17	0	0
{*s*_3_,*s*_7_,*s*_8_}	.09	0	0	0
{*s*_4_,*s*_7_,*s*_8_}	.09	.17	0	0
{*s*_1_,*s*_4_,*s*_8_}	.09	.17	.33	0
{*s*_1_,*s*_3_,*s*_7_,*s*_8_}	.09	0	0	0
{*s*_3_,*s*_4_,*s*_7_,*s*_8_}	.09	.17	0	0
{*s*_1_,*s*_4_,*s*_7_,*s*_8_}	.09	.17	.33	0
{*s*_1_,*s*_3_,*s*_4_,*s*_7_,*s*_8_}	.09	.17	.33	1

At step *m* = 1, according to the half-split questioning rule, problem *s*_4_ is selected because it minimizes the value of **Q**_*m*_ (see the second column of Table 2). Suppose that a correct response is obtained for this problem. After an application of the updating rule (Eq. 4), the likelihood of every knowledge state $K \in \mathcal {K}_{s_{4}}$ that contains problem *s*_4_ is ${\mathscr{L}}_{1}(K)=.17$ and that of a state $K^{\prime } \in \mathcal {K}_{\overline {s_{4}}}$ is ${\mathscr{L}}_{1}(K^{\prime })=.01$ (see the third column of Table 1).

At step *m* = 2, the problem that minimizes the half-split questioning rule is *s*_1_, as shown in the third column of Table 2. Suppose that the correct solution process (*s*_1_,*s*_3_,*s*_5_,*s*_7_,*s*_8_,*s*_9_) is observed for the problem. An application of the updating rule yields the likelihood distribution ${\mathscr{L}}_{2}$ which is shown in the fourth column of Table 1. The knowledge states in the intersection $\mathcal {K}_{s_{1}} \cap \mathcal {K}_{s_{4}}$ have a larger likelihood (i.e., .32) than that of every other knowledge state.

It should be noticed that the observed solution path of problem *s*_1_ contains those of both problems *s*_3_ and *s*_7_. According to the sub-path assumption, if *s*_1_ is contained in the knowledge state of the learner, both *s*_3_ and *s*_7_ must be contained in it. According to this assumption, a knowledge state containing all three problems *s*_1_, *s*_3_, and *s*_7_ should have a higher likelihood than a knowledge state that misses anyone of them. As can be seen from the fourth column of Table 1, this does not happen in the CMP. For instance, ${\mathscr{L}}_{2}{\{s_{1}, s_{4}, s_{8}\}}=.32 > .01 = {\mathscr{L}}_{2}{\{s_{1}, s_{3}, s_{7}, s_{8}\}}$. This shows that in the CMP there is no mechanism for exploiting the surplus of information that is made available by the observed solution process, and that a new updating rule is needed for this.

**Table 2:** Values of **Q**_*m*_ obtained during the Example 1 for each step *m*

*q*	*m* = 1	*m* = 2	*m* = 3
*s*_1_	.273	**.001**	1.000
*s*_3_	.273	.333	**.333**
*s*_4_	**.091**	.998	.999
*s*_7_	.273	.333	.334
*s*_8_	.818	1.000	1.000

The minimal values for **Q**_*m*_ at each step is in bold

To complete the example, one further question is required at step *m* = 3. The half-split questioning rule selects problem *s*_3_ (see the fourth column of Table 2). Suppose that a correct response is obtained. After the last update of the likelihood, the recovered knowledge state turns out to be {*s*_1_,*s*_3_,*s*_4_,*s*_7_,*s*_8_)}.

### Updating rules

The assessment procedures proposed in this article are capable of exploiting the whole observable solution process in updating the likelihood of the knowledge states. The assessment procedures consist of two nested loops. The outer loop starts with the presentation of a new problem (*s*_*m*,0_,*g*) ∈ *Q*, where *m* ≥ 1, whereas the inner loop starts with a new problem state *s*_*n*_ ∈ *S*, with *n* ≥ 0, in the solution process of (*s*_*m*,0_,*g*). For every new problem state *s*_*n*_ of the solution process of problem (*s*_*m*,0_,*g*) the likelihood distribution ${\mathscr{L}}_{m,n}$ is updated as follows:

9 $$ \mathcal{L}_{m,n+1}(K)=\frac{P(s_{m,n+1}|s_{m,n},s_{m,0},K) \mathcal{L}_{m,n}(K)}{{\sum}_{K^{\prime} \in \mathcal{K}}P(s_{m,n+1}|s_{m,n},s_{m,0},K) \mathcal{L}_{m,n}(K^{\prime})}, $$

where *P*(*s*_*m*,*n*+ 1_|*s*_*m*,*n*_,*s*_*m*,0_,*K*) is the conditional probability of the transition from *s*_*m*,*n*_ to *s*_*m*,*n*+ 1_, given knowledge state $K \in \mathcal {K}$ and problem (*s*_*m*,0_,*g*). It should be noted that Eq. 9 is nothing else than an adaptation of the Doignon and Falmagne’s Bayesian updating rule described in Eq. 2.

**Fig. 3:**
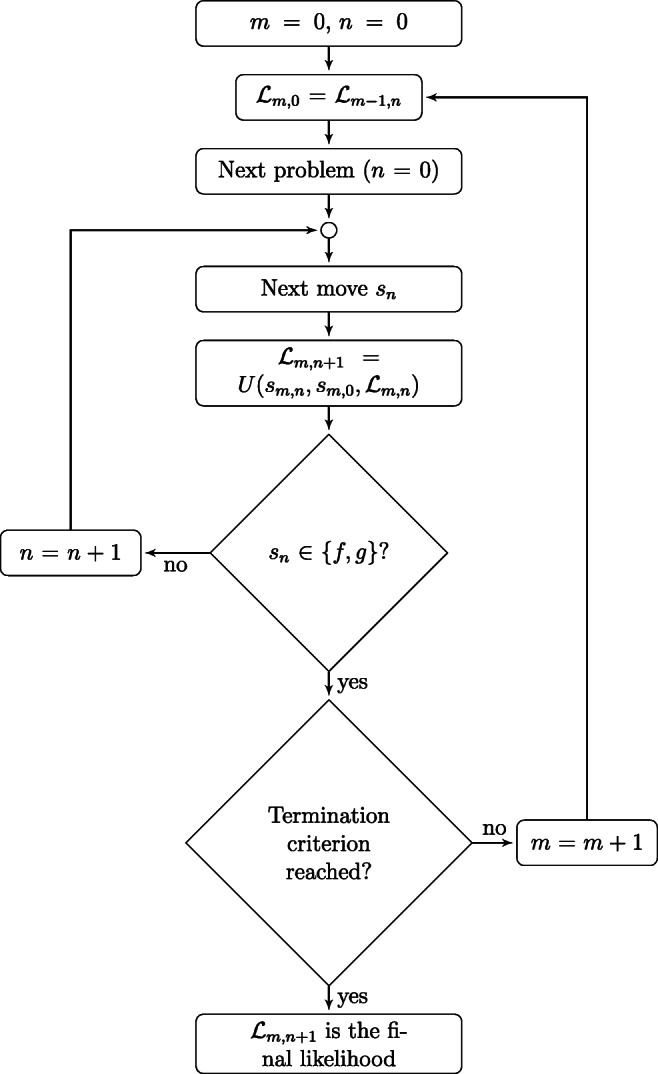
Diagram of the MSP-based procedure. See text for the details

As stated in Section “The Markov solution process model”, specific assumptions can be introduced on the conditional probability *P*(*s*_*m*,*n*+ 1_|*s*_*m*,*n*_,*s*_*m*,0_,*K*), for reducing the number of the parameters. One of these assumptions is (MSP1) described in Eq. 8. It is recalled that in this assumption the transition probability from *i* to *j* depends on the initial problem state *s*_0_ only. Two new assumptions denoted by (MSP2) and (MSP3) are presented below.

Assumption (MSP2) differs from (MSP1) from the fact that the transition probability from a problem state *i* to another problem state *j* is independent of the initial problem state *s*_0_. Under this assumption, for every problem (*s*_0_,*g*) ∈ *Q*, every pair (*i*,*j*) ∈ *E* and every knowledge state $K \in \mathcal {K}$ the transition probability is

10 $$ P(i|j,s_{0},K) = \begin{cases} \beta_{ij} & \text{if } i \in K;\\ \eta_{ij} & \text{if } i \notin K.\\ \end{cases} $$

Such probability is a *β*_*ij*_ parameter if problem *i* ∈ *K* belongs to the knowledge state $K \in \mathcal {K}$, it is a *η*_*ij*_ parameter otherwise.

According to assumption (MSP3), the transition probability from a problem state *i* to another problem state *j* depends on whether both problems *s*_0_ and *i* belong or not to the knowledge state $K \in \mathcal {K}$. For every problem (*s*_0_,*g*) ∈ *Q*, every pair (*i*,*j*) ∈ *E* and every knowledge state $K \in \mathcal {K}$ the transition probability is

11 $$ P(i|j,s_{0},K) = \begin{cases} \beta_{ij} & \text{if } s_{0} \in K \text{ and } i \in K;\\ \eta_{ij} & \text{if } s_{0} \notin K \text{ or } i \notin K.\\ \end{cases} $$

In particular, the probability of the transition from *i* to *j* is a *β*_*ij*_ parameter if the individual knows at least one solution path for both problems *s*_0_ and *i*. Otherwise, the transition probability is a *η*_*ij*_ parameter.

The three different assumptions are plausible in different situations. The MSP1 assumption is plausible when a problem solver plans ahead the whole solution process of the problem and every single move sticks to the initial plan. For this reason, (MSP1) can be regarded as a *pre-planning assumption*. On the other side, the MSP2 assumption allows interim planning. It might well be that an initial plan is built, however this last may change along the way. Thus, the transition from a problem state *i* to another one depends on problem state *i* only. For this reason, (MSP2) can be regarded as an *interim-planning assumption*. Finally, according to assumption MSP3, a correct solution to the problem depends on both the initial (*s*_0_) and current (*i*) problem states. In particular, any transition probability is a *β* if and only if both *s*_0_ and *i* belong to the knowledge state. In this sense, (MSP3) combines together MSP1 and MSP2 like an “AND” Boolean operator on the *β*_*ij*_. Given this interpretation, (MSP3) can be named as a *mixed planning assumption*.

**Table 3:** Summary of the parameters obtained under the three assumptions

(*s*_0_,*g*) ∈ *K*	(*i*,*g*) ∈ *K*	MSP1	MSP2	MSP3
yes	yes	*β*	*β*	*β*
yes	no	*β*	*η*	*η*
no	yes	*η*	*β*	*η*
no	no	*η*	*η*	*η*

Columns 1 and 2 display, respectively, if the initial problem and the current problem belong to the considered knowledge state. Columns 3–5 display the resulting parameters under that assumption

Table 3 summarizes the parameters obtained by the three assumptions in function of the initial and the current problem states (columns 1 and 2 in the table). For instance, if *s*_0_ ∈ *K* and *i*∉*K* (Row 3 in the table), under assumption MSP1 the transition probability from *i* to *j* is a *β*_*ij*_ parameter whereas under assumptions MSP2 and MSP3 the same transition is an *η*_*ij*_ parameter. It is worth mentioning that other assumptions are possible like, for instance, one that behaves like a Boolean operators “OR” on the *β*_*ij*_. However, such assumptions are not considered in this research. When applied to the MSPM, the three different assumptions MSP1, MSP2, and MSP3 gives rises to three different models, henceforth named MSPM1, MSPM2, and MSPM3, respectively.

### Procedures based on the Markov solution process model

In this section, an MSP-based adaptive assessment procedure is presented that is based on the updating rule shown in Eq. 9. It is worth noticing that this procedure can be applied with any of the MSP1, MSP2, and MSP3 assumptions (and it is open to other assumptions).

Figure 3 illustrates the flowchart of the procedure. The assessment procedure consists of two nested loops. The outer loop starts with the presentation of a new problem (*s*_*m*,0_,*g*) ∈ *Q*, where *m* ≥ 1, whereas the inner loop starts with a new problem state *s*_*m*,*n*_ ∈ *S*, with *n* ≥ 0, in the solution process of the problem (*s*_*m*,0_,*g*).

At the beginning of the assessment (i.e., *m* = 0 and *n* = 0), the likelihood ${\mathscr{L}}_{0,0}$ is set to be a uniform distribution among the knowledge states. Starting from ${\mathscr{L}}_{0,0}$, the assessment is carried out in an iterative way. At each step *m*, the likelihood ${\mathscr{L}}_{m,0} ={\mathscr{L}}_{m-1,n}$ and a problem (*s*_*m*,0_,*g*) ∈ *Q* is selected according to the questioning rule. In this work, the half-split questioning rule presented in Eq. 1 has been used. The updating rule described by Eq. 9 obtains ${\mathscr{L}}_{m,n+1} $ from the ${\mathscr{L}}_{m,n} $ given that the current problem is (*s*_*m*,0_,*g*) and the observed problem state in the solution process is *s*_*m*,*n*+ 1_. The solution process for problem (*s*_*m*,0_,*g*) terminates whenever the observed problem state *s*_*m*,*n*+ 1_ is the goal state *g* or the failure state *f*. The termination criterion decides whether an additional problem should be presented or not. The assessment terminates as soon as the likelihood ${\mathscr{L}}_{m,n+1} (K) $ of any knowledge state $K \in \mathcal {K}$ is greater than a predefined value *p* ∈ (.5,1].

**Table 4:** Values of the *β*_*ij*_ and *η*_*ij*_ parameters used in the Example 2

*i*	*j*	*β*_*ij*_	*η*_*ij*_
*s*_1_	*s*_2_	0.36	0.01
*s*_1_	*s*_3_	0.62	0.03
*s*_2_	*s*_4_	0.99	0.06
*s*_3_	*s*_5_	0.99	0.08
*s*_4_	*s*_6_	0.99	0.01
*s*_5_	*s*_7_	0.99	0.09
*s*_6_	*s*_8_	0.99	0.09
*s*_7_	*s*_8_	0.99	0.09
*s*_8_	*s*_9_	0.99	0.08
*s*_9_	*s*_9_	1.00	1.00

#### *Example 2*

Consider the problem space depicted in Fig. 2 and the knowledge space $\mathcal {K}_{\text {ToL}}$ derived in the running example in Section “Procedural knowledge space theory”. Suppose that the MSP-based procedure, with the mixed-planning assumption, is applied to uncover the knowledge state {*s*_1_,*s*_3_,*s*_4_,*s*_7_,*s*_8_} of the same problem solver introduced in Example 1.

Table 4 shows the *β*_*ij*_ (third column) and *η*_*ij*_ (fourth column) assumed in this example. In particular, each row of the columns shows the transition probabilities from the problem state *i* (first column) to the problem state *j* (second column). The transition probabilities to the failure state are obtained as

$$ \beta_{if} = 1-\sum\limits_{j \in S_{\text{ToL}} \setminus \{f\}} \beta_{ij} \quad\quad \text{ and } \quad\quad \eta_{if} = 1-\sum\limits_{j \in S_{\text{ToL}} \setminus \{f\}} \eta_{ij}. $$

At the beginning of the assessment (i.e., *m* = 0 and *n* = 0), all of the knowledge states $K \in \mathcal {K}_{\text {ToL}}$ have the same likelihood ${\mathscr{L}}_{0}(K)= 1/ |\mathcal {K}_{\text {ToL}}|$ (see the second column of Table 5).

**Table 5:** Values of the likelihood distribution ${\mathscr{L}}_{m}$ at each step *m* of the assessment procedure

*K*	$ {\mathscr{L}}_{0}(K)$	$ {\mathscr{L}}_{1}(K)$	$ {\mathscr{L}}_{2}(K)$
{*∅*}	.09	0	0
{*s*_8_}	.09	0	0
{*s*_7_,*s*_8_}	.09	0	0
{*s*_4_,*s*_8_}	.09	.17	0
{*s*_3_,*s*_7_,*s*_8_}	.09	0	0
{*s*_4_,*s*_7_,*s*_8_}	.09	.17	0
{*s*_1_,*s*_4_,*s*_8_}	.09	.17	.01
{*s*_1_,*s*_3_,*s*_7_,*s*_8_}	.09	0	0
{*s*_3_,*s*_4_,*s*_7_,*s*_8_}	.09	.17	0
{*s*_1_,*s*_4_,*s*_7_,*s*_8_}	.09	.17	.07
{*s*_1_,*s*_3_,*s*_4_,*s*_7_,*s*_8_}	.09	.17	.92

According to the half-split questioning rule, at step *m* = 1 and *n* = 0 problem *s*_4_ is selected because it minimizes the value of **Q**_*m*_ (see the second column of Table 2). Suppose that at step *m* = 1 and *n* = 3 the correct solution process (*s*_4_,*s*_6_,*s*_8_,*s*_9_) is observed for problem *s*_4_. After three updates of the likelihood distribution (one for each move), the likelihood of every knowledge state *K* that contains both problems *s*_4_,*s*_8_ is ${\mathscr{L}}_{1}(K)=.17$, whereas that of every knowledge state $K^{\prime }$ containing neither *s*_4_ nor *s*_8_ is ${\mathscr{L}}_{1}(K^{\prime })=0$ (see the third column of Table 5).

At step *m* = 2 and *n* = 0, the half-split questioning rule selects problem *s*_1_, as shown in the third column of Table 2. Suppose that the correct solution process (*s*_1_,*s*_3_,*s*_5_,*s*_7_,*s*_8_,*s*_9_) is observed for the problem. At the last sub-step *n* = 5, the likelihood was updated five times and the knowledge state {*s*_1_,*s*_3_,*s*_4_,*s*_7_,*s*_8_} obtained the largest likelihood, as shown in the third column of Table 5. This was also the last question asked by the procedure because the maximum likelihood exceeded the termination criterion of .5. Thus, the MSP-based procedure inferred the knowledge state of the problem solver in two questions out of five. Comparing this example with Example 1, it can be noticed that the MSP-based procedure is more efficient than the CMP, even in this trivial example. Indeed, the CMP requires one more question to terminate. This is because the proposed procedure exploits the fact that according to the sup-paths assumption, if *s*_1_ is contained in the knowledge state of the problem solver, both *s*_3_ and *s*_5_ must be contained.

To show that a latent knowledge state $K_{0} \in \mathcal {K}$ is uncoverable by the MSP-based procedures, it suffices to assure that the updating rule in Eq. 9 is multiplicative.

#### **Theorem 1**

The updating rule in Eq. 9 is multiplicative if and only if for all the transitions (*i*,*j*) ∈ *E*, *β*_*ij*_ > *η*_*ij*_ and *η*_*if*_ > *β*_*if*_.

#### *Proof*

Let *ι*_*K*_(*s*) be the indicator function of *K*, which is defined on *Q* by

12 $$ \iota_{K} (s) = \begin{cases} 1 & \text{if } (s,g) \in K \\ 0 & \text{if } (s,g) \in Q \setminus K.\\ \end{cases} $$

Moreover, for *i* ∈ *S*, let *E*(*i*) = {*j* ∈ *S* : (*i*,*j*) ∈ *E*}, and define the function $R:E(i) \rightarrow \{0,1\}$ such that for each *j* ∈ *E*(*i*)

13 $$ R(j) = \begin{cases} 1 & \text{if } j\neq f \\ 0 & \text{if } j = f.\\ \end{cases} $$

We are aimed at showing that the following equality holds true:

14 $$ \begin{array}{@{}rcl@{}} &&\frac{P(s_{m,n+1}|s_{m,n},s_{m,0},K) \mathcal{L}_{m,n}(K)}{{\sum}_{K^{\prime} \in \mathcal{K}}P(s_{m,n+1}|s_{m,n},s_{m,0},K) \mathcal{L}_{m,n}(K^{\prime})}\\ &&\quad=\frac{\zeta_{q,r}^{K} \mathcal{L}_{m,n}(K)}{{\sum}_{K^{\prime} \in \mathcal{K}}\zeta_{q,r}^{K^{\prime}} \mathcal{L}_{m,n}(K^{\prime})}. \end{array} $$

For (*i*,*j*) ∈ *E* and *s*_0_ ∈ *Q*_*g*_ let

15 $$  \zeta_{s_{0},i,j}=\frac{\beta_{ij}}{\eta_{ij}} \text{ and } \zeta_{s_{0},i,f}=\frac{\eta_{if}}{\beta_{if}}. $$

There are four cases. Case 1 is *ι*_*K*_(*s*_0_) = 1 and *R*(*j*) = 1. In this case Eq. 14 becomes:

$$ \begin{array}{@{}rcl@{}} &&\frac{P(s_{m,n+1}|s_{m,n},s_{m,0},K) \mathcal{L}_{m,n}(K)}{{\sum}_{K^{\prime} \in \mathcal{K}}P(s_{m,n+1}|s_{m,n},s_{m,0},K) \mathcal{L}_{m,n}(K^{\prime})}\\ &&\quad=\frac{\zeta_{s_{0},i,j} \mathcal{L}_{m,n}(K)}{ \zeta_{s_{0},i,j} \mathcal{L}_{m,n}(\mathcal{K}_{s_{0}}) + \mathcal{L}_{m,n}(\mathcal{K}_{\overline{s_{0}}}) } \end{array} $$

By applying Eq. 15, the right-hand side term of the equation becomes:

$$ \begin{array}{@{}rcl@{}} \frac{\beta_{ij}/\eta_{ij} \mathcal{L}_{m,n}(K)}{\beta_{ij}/\eta_{ij} \mathcal{L}_{m,n}(\mathcal{K}_{s_{0}}) + \mathcal{L}_{m,n}(\mathcal{K}_{\overline{s_{0}}})}, \end{array} $$

which then gives

$$ \begin{array}{@{}rcl@{}} \frac{\beta_{ij} \mathcal{L}_{m,n}(K)}{\beta_{ij} \mathcal{L}_{m,n}(\mathcal{K}_{s_{0}}) + \eta_{ij}\mathcal{L}_{m,n}(\mathcal{K}_{\overline{s_{0}}})}, \end{array} $$

which turns out to be the MSP-based updating rule for Case 1. We omit the proof for each of the remaining three cases: Case 2, *ι*_*K*_(*s*_0_) = 1 and *R*(*j*) = 0; Case 3, *ι*_*K*_(*s*_0_) = 0 and *R*(*j*) = 1; and Case 4, *ι*_*K*_(*s*_0_) = 0 and *R*(*j*) = 0, because they can be trivially obtained by applying the obvious substitutions. □

## Simulation study

The aim of the study was to compare to one another the three adaptive procedures based on MSP1, MSP2, and MSP3 assumptions. Moreover, the performance of the three procedures was compared with that of the more known and used CMP. The comparison was made in terms of efficiency and accuracy.

### Goal spaces of the Tower of London

The assessment procedures described in this research are for general purpose, as long as procedural assessment of knowledge is concern. In the following studies, they are applied to the case of the ToL test. Since the problem is correctly solved only if the solution is obtained with a minimum number of moves, the goal space of the ToL happens to be a special case called *shortest path space* (SP space; Stefanutti et al., 2021). Such a type of goal spaces can often arise in applications like the ToL. Further considerations and properties of the SP spaces as well as the accurate description of the goal spaces and knowledge spaces used in this application can be found in Stefanutti et al., (2021).

**Fig. 4:**
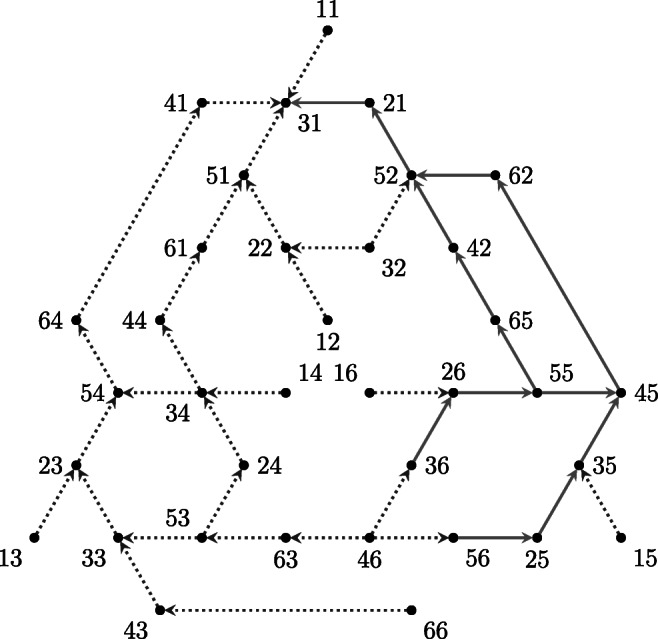
The two goal spaces $\mathbf {P}_{g}^{(1)}$,and $\mathbf {P}_{g}^{(2)}$ used in both simulation studies. It is recalled that both goal spaces are shortest paths spaces

The goal spaces considered in this study were obtained by setting problem state 31 as the goal state (see Fig. 4).

The goal space $\mathbf {P}_{g}^{(1)}$ is represented in Fig. 4 using solid lines. Such goal space is composed by 12 problem states plus the goal and the failure states. The number of problems involved in such a goal space is 12. One of them was removed because of its easiness (only one move was required to solve it). Thus, the domain $Q_{g}^{(1)}$ of the goal space $\mathbf {P}_{g}^{(1)}$ contains 11 problems, three of them having two alternative solutions. The other goal space $P_{g}^{(2)}$ used in this study was obtained from the problem space by setting problem state 31 as the goal state (both dotted and solid line in Fig. 4). The set of problems involved in this goal space is 35, however all problems requiring only one move were removed. Thus, the set of problems $Q_{g}^{(2)}$ of this goal space is 31, 11 of them having more than one solutions. The two goal spaces delineate two knowledge spaces $\mathcal {K}_{1}$ with 61 knowledge states and $\mathcal {K}_{2}$ with 242,498 knowledge states.

**Table 6:** Design of the simulation study used for generating the data

Cond.	Model	$|\mathcal {K}|$	Error	*N*
1	MSPM1	61	.01	155
2	MSPM1	61	.01	1000
3	MSPM1	61	.20	155
4	MSPM1	61	.20	1000
5	MSPM2	61	.01	155
6	MSPM2	61	.01	1000
7	MSPM2	61	.20	155
8	MSPM2	61	.20	1000
9	MSPM3	61	.01	155
10	MSPM3	61	.01	1000
11	MSPM3	61	.20	155
12	MSPM3	61	.20	1000
13	MSPM1	242,498	.01	1000
14	MSPM1	242,498	.01	100,000
15	MSPM1	242,498	.20	1000
16	MSPM1	242,498	.20	100,000
17	MSPM2	242,498	.01	1000
18	MSPM2	242,498	.01	100,000
19	MSPM2	242,498	.20	1000
20	MSPM2	242,498	.20	100,000
21	MSPM3	242,498	.01	1000
22	MSPM3	242,498	.01	100,000
23	MSPM3	242,498	.20	1000
24	MSPM3	242,498	.20	100,000

Column 1 displays the condition number, column 2 displays the assumption underlying the data generation. Column 3 displays which knowledge space was used. Column 4 displays the maximum amount of error used for generating the data and column 5 displays the sample size

### Simulation design and data set generation

Table 6 shows the simulation design used for generating the data sets.

The manipulated variables were: (i) the generative model, that could be the MSPM1, the MSPM2, or the MSPM3; (ii) the true knowledge structure, that could be $\mathcal {K}_{1}$ composed by 61 states, or $\mathcal {K}_{2}$, composed by 242,498 states; (iii) the amount of error in the data, that was at maximum .01 or .20; and (iv) the sample size *N*, that could be 155 or 1000 when the considered structure was $\mathcal {K}_{1}$ and 1000 and 100,000 when the structure was $\mathcal {K}_{2}$.

Concerning with knowledge structures, the choice was to use one feasible structure ($\mathcal {K}_{1}$) and one huge structure ($\mathcal {K}_{2}$). The former was the structure derived from the goal space $\textbf {P}_{g}^{(1)}$. This structure has also been considered for collecting real data that were used in the study presented in “Simulation study based on real data”. The latter structure was derived from the goal space $\textbf {P}_{g}^{(2)}$.

As for the “amount of error” in the data, it has been manipulated through the two types of parameters *β*_*ij*_ and *η*_*ij*_ that are present in all three models. The values of these parameters used for generating the data were exactly the same for all models. They have been generated in the following way. For *i* ∈ *S*_*g*_ ∖{*f*,*g*}, first the probabilities *β*_*if*_ and *η*_*if*_ were extracted at random from a uniform distribution in the interval (0;*x*] and (0;1 − *x*], where *x* ∈{.01,.20}, respectively. These two intervals have been chosen in order to have a situation of a very small error in the data (the former case), and a situation of a large error in the data (the latter case). We recall, in fact, that *β*_*if*_ is interpreted as a careless error probability, and, for *i*≠*f*, *η*_*ij*_ is interpreted as a lucky guess probability. Then, the probabilities *β*_*ij*_ and *η*_*ij*_, with *i*≠*j*, were generated at random from a uniform distribution in the interval (0,1), and then normalized to sum up to 1 − *β*_*ij*_ and 1 − *η*_*ij*_, respectively.

In the whole, a 3 × 2 × 2 × 2 = 24 different conditions have been considered and, in each of them, one sample was generated. The procedure used for generating the samples is described below.

Each simulated response pattern corresponded to a collection of *J*_*q*_ jump matrices, one for each item *q* ∈ *Q*_*g*_. Moreover, every single “simulated subject” is represented by a pair (*J*,*K*), where *K* is a knowledge state and *J* is a response pattern. In the sequel, the response pattern *J* is referred to as the “response pattern generated by the true state *K*”.

For generating the pair (*J*,*K*) the procedure started with the extraction of *K* from the knowledge structure $\mathcal {K}$, with a certain probability. More precisely, for each state $K^{\prime } \in \mathcal {K}$, a random number was extracted from a uniform distribution in the (0,1) interval. A set of values was obtained that was normalized to sum up to 1. In this way, a random probability distribution $\pi _{\mathcal {K}}$ was generated, which determined the extraction probability of each state. The knowledge states extracted at each iteration and the probability distribution $\pi _{\mathcal {K}}$ were kept fixed across the different conditions 1 to 12, when the true knowledge structure was $\mathcal {K}_{1}$, and across 13 to 24 conditions, when the true knowledge structure was $\mathcal {K}_{2}$.

Given knowledge state *K*, the response pattern *J* was obtained as follows. For each item *q* ∈ *Q*_*g*_, a sequence of moves

$$ J_{q}=(s_{1},s_{2},\ldots,s_{i},\ldots,s_{n}) $$

was generated. Such sequence was obtained iteratively, as explained below. For each *i* ∈{1,2…,*n* − 1}, problem state *s*_*i*+ 1_ was randomly generated under different rules, depending on the generative model. Under model MSPM1, $P(s_{i+1}|s_{i},s_{1},K)=\beta _{s_{i},s_{i+1}}$ whenever (*s*_1_,*g*) ∈ *K*, and $P(s_{i+1}|s_{i},q,K)=\eta _{s_{i},s_{i+1}}$ whenever (*s*_1_,*g*) ∈ *Q*_*g*_ ∖ *K*. Under model MSPM2, $P(s_{i+1}|s_{i},s_{1},K)=\beta _{s_{i},s_{i+1}}$ whenever (*s*_*i*_,*g*) ∈ *K*, and $P(s_{i+1}|s_{i},q,K)=\eta _{s_{i},s_{i+1}}$ whenever (*s*_*i*_,*g*) ∈ *Q*_*g*_ ∖ *K*. Under model MSPM3, $P(s_{i+1}|s_{i},s_{1},K)=\beta _{s_{i},s_{i+1}}$ whenever $\{(s_{1},g),(s_{i},g)\} \subseteq K$, and $P(s_{i+1}|s_{i},q,K)=\eta _{s_{i},s_{i+1}}$ whenever $\{(s_{1},g),(s_{i},g)\} \not \subseteq K$.

For each item, the iterations terminated when one of the two problem states *f* (failure) or *g* (goal) was entered. It is worth noticing that the termination of each iteration was assured by the fact that $\mathbf {P}_{g}^{(1)}$ and $\mathbf {P}_{g}^{(2)}$ were goal spaces.

This procedure was applied iteratively until *N* pairs (*J*,*K*) were obtained for each generative model. In the end, three types of data were obtained, that is *D*_1_, generated under the MSPM1, *D*_2_, generated under the MSPM2, and *D*_3_, generated under the MSPM3.

With the aim of applying the CMP adaptive procedure, the simulated response patterns belonging to *D*_1_, *D*_2_, and *D*_3_ have been “dichotomized” obtaining data set *D*_4_. For each problem, only the accuracy (correct vs. incorrect) was considered. More in detail, if (*s*_1_,…,*s*_*n*_) represents the observed sequence of moves for problem *q*, then the “dichotomous” answer to *q* was marked as “correct” if *s*_*n*_ = *g* and as “incorrect” if *s*_*n*_ = *f*.

### Methods

The procedures based on MSP1, MSP2, and MSP3 were applied to each of the 24 samples (one sample per simulation condition). Moreover, the dichotomous version of each sample was used with the CMP’s adaptive procedure. Thus, each sample was used with four different procedures.

All the four adaptive procedures were applied to the simulated response patterns in the following way. Let *w* ∈{1,2,…,*N*}, and let *J*^*w*^ denote the *w*-th simulated subject. For each *J*^*w*^, each step *m* of the assessment, with *m* ∈{1,2,…,|*Q*_*g*_|}, consisted of *m* updating of the knowledge states likelihood ${\mathscr{L}}(m)$. This updating depended on the response to problem *q* selected by the procedure at that step. Thus, *m* increased with the number of problems asked and not with state transitions. The response to problem *q* was stored in advance in the simulated samples *D*_1_, *D*_2_, *D*_3_, and *D*_4_, respectively when the adaptive procedure based on the MSP1, the MSP2, the MSP3, and the CMP were considered.

At each step *m* of a particular procedure, the modal knowledge state $\widehat {K}^{w}_{m}$ of the simulated subject *J*^*w*^ was estimated. The estimation procedure consisted of taking the state $K \in \mathcal {K}$ for which the likelihood ${\mathscr{L}}_{m}^{w}$ was maximum. When $\max \limits ({\mathscr{L}}_{m}^{w})>.50$, then a unique $\widehat {K}^{w}_{m}$ existed, otherwise the modal knowledge state may be not unique. In such a case, the only way for assigning a knowledge state to a subject is a random choice among the modal states.

For each condition of the simulation design, the accuracy and the efficiency of the procedures have been analyzed at each step *m* of the assessment by using several performance indexes.

#### Performance accuracy indexes

Concerning the accuracy, two performance indexes have been considered for each procedure, that is:

1. The average Hamming distance $\bar {D}_{m}(K^{w},\hat {K}_{m}^{w})$ computed by
  16 $$ \bar{D}_{m}(K^{w},\hat{K}_{m}^{w})=\frac{1}{N}\sum\limits_{w=1}^{N} |K^{w} {\Delta} \hat{K}_{m}^{w}|, $$
  where Δ represents the symmetric set difference.
2. The true-positive rate TPR computed at the end of the assessment, that is the proportion of pairs (*J*^*w*^,*K*^*w*^) for which $K^{w} = \hat {K}_{m}^{w}$, with *m* = |*Q*_*g*_|.

#### Performance efficiency indexes

The efficiency of each procedure was measured by three indexes. For each participant *w*, the number of problems asked *m*^*w*^ until the termination criterion ${\mathscr{L}}_{m}^{w}(\hat {K}_{m}^{w})>.50$ is reached was registered. This index has a frequency distribution in the simulated data set, having the set {1,2,⋯ ,|*Q*_*g*_|} as a support. Two of the three efficiency indexes considered in this study were the mean $\bar {m}$ of this distribution and its cumulative distribution.

The last index was the Shannon’s entropy (Shannon, 1948). This metric is used in information theory for quantifying the “amount of information” contained in a variable, in terms of the number of bits it takes to store the variable. In the context of computing the efficiency of an adaptive assessment procedure, this metric informs on how many “bits of information” are missing for having the maximal information on the whole test. Each bit of information is an item of the test. It was computed as

$$ {H^{w}_{m}}=-\sum\limits_{K \in \mathcal{K}} \mathcal{L}^{w}_{m}(K) \log_{2} \mathcal{L}^{w}_{m}(K). $$

The average $\bar {H}_{m}$ of this quantity was computed across all simulated subject for each number *m* of questions asked.

### Results

#### Accuracy

**Fig. 5:**
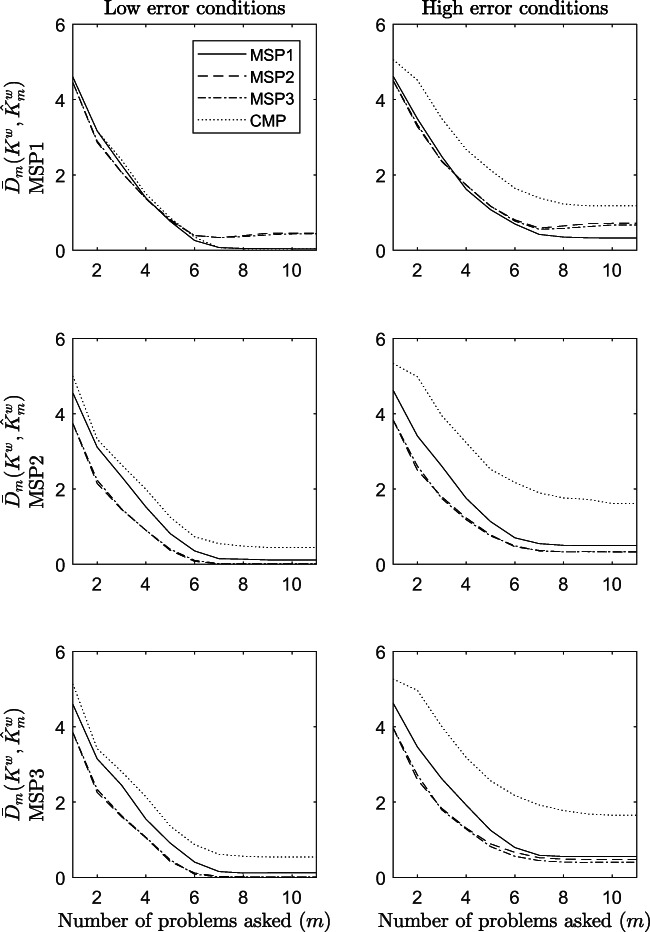
Accuracy of the procedures in terms of average Hamming distance between the true and the estimated knowledge state. The results refer to odds conditions from 1 to 12 of the simulation study

Figure 5 shows the results obtained on the accuracy of the procedures when the average Hamming distance is used as the performance index, and $\mathcal {K}_{1}$ is the considered knowledge structure. In the figure, panels to the left refer to conditions in which the maximum amount of error in the data was .01 (named, in the figure, low error conditions). Panels to the right refer to simulation conditions in which the maximum amount of error in the data was .20 (named high error conditions). Row panels refer to the model used for generating the data, which is MSPM1, MSPM2, and MSPM3, respectively, from the top to the bottom of the figure. In each panel, the number *m* of problems asked by a procedure is along the *x*-axis, and the average Hamming distance $\bar {D}_{m}(K^{w},\hat {K}_{m}^{w})$ is along the *y*-axis. The smaller the distance, the better the performance.

As expected by an adaptive assessment procedure, the average Hamming distance decreases as the number of questions asked increases. This is true for all procedures, irrespective of the amount of error in the data, and of the generative model. Another quite evident result is that among the four procedures, the CMP is the one most susceptible to noise. Indeed, the difference in the performance between conditions with low error in the data and conditions with high error is the greatest for this model.

As for the other models, the effect of the amount of error in the data can be seen in the values of $\bar {D}_{m}(K^{w},\hat {K}_{m}^{w})$ reached by the procedures at each step *m* of the assessment and, mostly, at the end (*m* = 11). Indeed, for all procedures, irrespective of the generative model, in conditions with low error in the data (panels to the left), the average Hamming distance is lower than that in conditions with a high error in the data (panels to the right). It approaches 0 only when the amount of error in the data is very low, but with a different extent depending on the generative model.

Interestingly enough, when the generative model is the MSPM1, in the low-error condition, both the MSP1 and the CMP procedures terminate with a distance $\bar {D}_{11}(K^{w},\hat {K}_{11}^{w})=0$, whereas the other two procedures had a slightly worse performance. A different result is obtained when the generative model is the MSPM2 or the MSPM3. Indeed, in these conditions, $\bar {D}_{11}(K^{w},\hat {K}_{11}^{w})$ reaches zero with the MSPM1, MSPM2, and MSPM3 models, whereas it is higher for the CMP.

The effect of the sample size on the Hamming distance is negligible (see Fig. 1 in the supplementary material of the article).

The results on the Hamming distance between the true state *K* and the estimated state $\hat {K}_{m}$ are better understood if considered along with the true-positive rate.

**Fig. 6:**
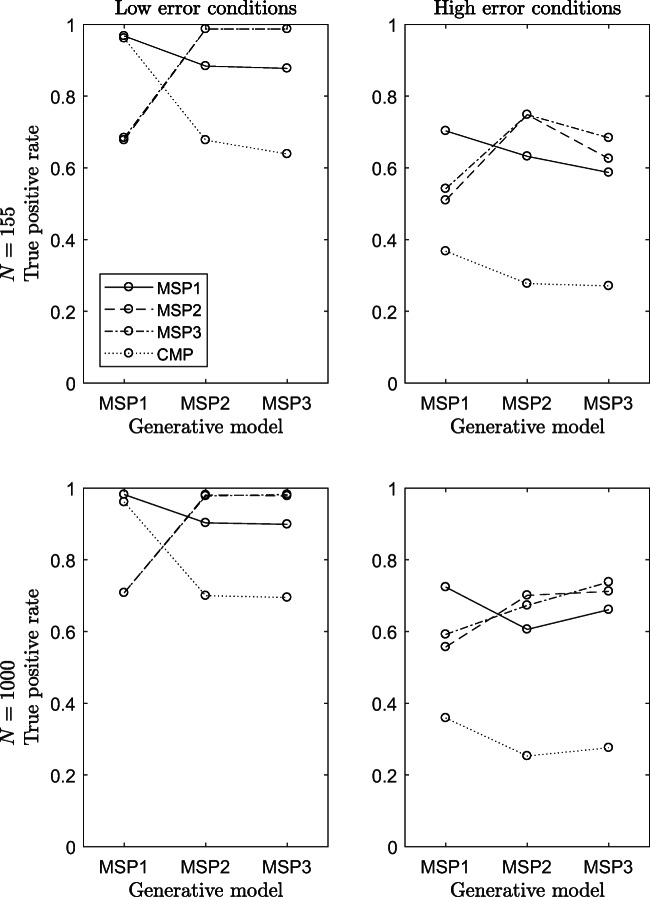
Accuracy of the procedures in terms of TPR in all conditions 1 to 12 of the simulation study

Figure 6 displays the results of the procedures’ accuracy in terms of true-positive rate, when the knowledge structure was $\mathcal {K}_{1}$. Panels to the top refer to conditions in which the sample size was *N* = 155 and those to the bottom refer to conditions with *N* = 1000. In each panel, the three generative models are along the *x*-axis and the true-positive rate is along the *y*-axis.

What clearly results is that the TPR of the CMP-based procedure is almost always lower than that of the MSP-based procedures. Its performances are equally good compared to those of the MSP1 and higher than those of the MSP2 and MSP3 only in two conditions of the simulation design out of 24, that is when the generative model is the MSPM1 and the amount of error in the data is low. Not surprisingly, these two conditions are very favorable for the CMP.

In conditions with low error in the data (panels to the left in Fig. 6), MSP2- and MSP3-based procedures perform equally well, reaching a TPR = 1.00 when they are the generative models. Instead, their performances are worse than those of the other two procedures when the generative model is the MSP1. In conditions with high error in the data (panels to the right), the performances of all procedures worsen. In these conditions, the CMP is able of finding the true knowledge state of the patterns only in a number of cases smaller than 50%.

Also for the TPR, it seems that the effect of the sample size on the procedure’s accuracy is negligible. Indeed, the bottom panels of Fig. 6 are almost the same as those to the top.

Reading jointly the results on the Hamming distance and the TPR, some interesting insights emerge about the efficiency of procedures when they are applied in a condition in which they are not the generative model. If the generative model is the MSPM1, both the MSPM2 and the MSPM3 procedures perform very well in terms of Hamming distances (their performances are very similar to those of the MSP1) but they perform less well in terms of TPR (they performances are about 20% worse than that of the MSP1), whereas when the generative model is the MSPM2 or the MSPM3, the performance of the MSP1 is quite good in terms of TPR (its performance is about 10% worse than the other two) but it is worse in terms of the Hamming distance. Thus, it seems that although the MSP2 and MSP3 procedures have a lower TPR than the MSP1 (they fail more often) they estimate a knowledge state that is closer to the true one in terms of Hamming distance.

Concerning Conditions 13 to 24, where the knowledge structure $\mathcal {K}_{2}$ having 242,498 states was used, very similar results of those described above (panels on the left of Figs. 3 and 6 in the supplementary material). In these conditions, the only obvious differences are in the values of the performance indexes reached by the procedures. In fact, the domain of $\mathcal {K}_{2}$ was composed by 31 problems (versus the 11 problems belonging to the domain of $\mathcal {K}_{1}$). The increasing of the number of problems affects, necessarily, both the accuracy and the efficiency of the procedures. Nevertheless, in proportion, the results are almost the same for all the performance indexes.

#### Efficiency

**Fig. 7:**
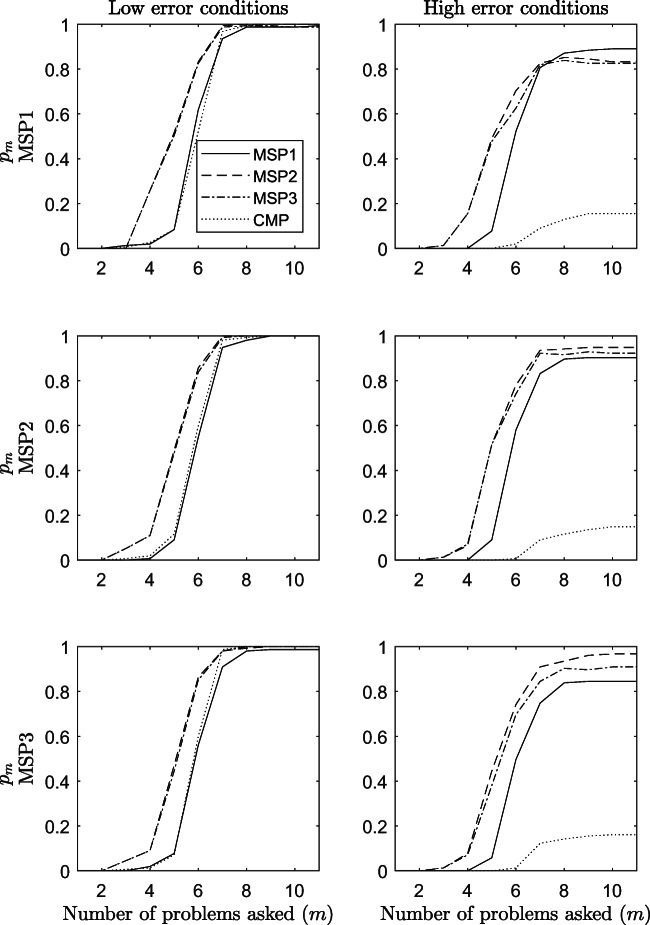
Efficiency of the procedures in terms of proportion of subjects that reached the termination criterion *p* ≥ .50 at step *m*. The results refer to odds conditions from 1 to 12 of the simulation study

Figure 7 shows the results on the efficiency of the procedures in terms of proportion of subjects *p*_*m*_ (*y*-axis) that reached the termination criterion ${\mathscr{L}}_{m}^{w}(\hat {K}_{m}^{w}) \ge .50$ at a particular step *m* (*x*-axis) of the assessment. The results refer to conditions with low error in the data (panels to the left) and with high error in the data (panels to the right), when the sample size is 155 and the structure is $\mathcal {K}_{1}$.

Interestingly enough, MSP2 and MSP3 perform better than the MSP1 and the CMP in almost all conditions, irrespective of the generative model and the amount of error in the data. In conditions with low error in the data, a proportion of simulated subjects greater than 80% reaches the termination criterion with MSP2 and MSP3 only after five questions, even when they are not the generative model. For the other two models, at least one more question is needed for arriving at the same proportion of the sample. It is worth noticing that in conditions with high errors in the data, the performance of the CMP is a lot worse. Indeed, less than the 20% of the sample reaches the termination criterion at the end of the assessment. At the end of the assessment, the other three procedures approach 100% of the sample when the amount of error is small, and a percentage greater than 80% when it is high. The effect of the sample size on this efficiency index is negligible (see Fig. 2 in the supplementary material of the article).

**Fig. 8:**
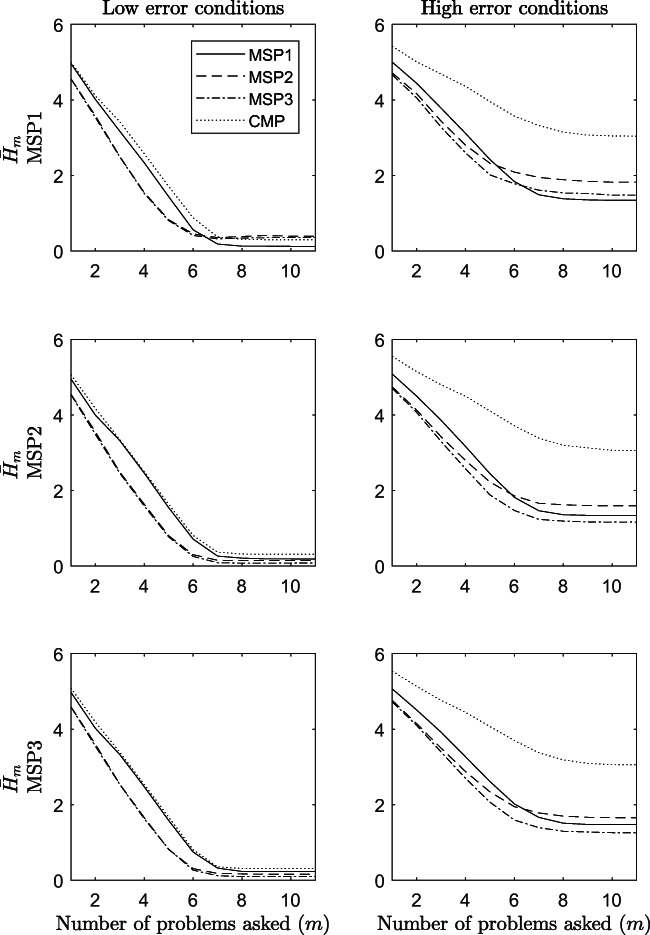
Efficiency of the adaptive procedures in terms of average entropy $\bar {H}_{m}$ at each step *m* of the assessment. The results refer to odds conditions from 1 to 12 of the simulation study

Concluding, the efficiency in terms of average entropy ${H^{w}_{m}}$ of the adaptive procedures is displayed in Fig. 8. The figure is read exactly like Fig. 5, with the only difference that along the *y*-axis, the average entropy $\bar {H}_{m}$ is displayed.

It can be seen that this index monotonically decreases as the number of problems asked increases. This is true irrespective of the generative model and the amount of error in the data. What emerges very clearly is that when the amount of error in the data was high (panels to the right), the procedure based on the CMP performed worse than the other three in all conditions. When the amount of error in the data was low (panels to the left), the CMP and the MSP1 performed very similarly one to another but worse than the MSP2 and the MSP3 procedures. Thus, also this statistic suggests that the MSP2 and the MSP3 procedures are more efficient than the other two.

Concerning Conditions 13 to 24, where the knowledge structure $\mathcal {K}_{2}$ having 242,498 states was used, the entropy show acceptable results (Figs. 4 and 7 of the supplementary material), however the proportion of subject that react the termination criteria (*p*_*m*_ ≥ .5) is rather poor when the error is high (right panel in Figs. 5 and 8 of the supplementary material). This could be due to the interaction between two factors, namely the huge size of the knowledge space and the high error level used in the simulation. In these conditions, a likelihood as large as .5 would hardly be reached by any assessment procedure. Maybe in a situation like this, such criterion is too strong and could be replaced by a weaker one, like the following: stop whenever a single modal state is obtained.

### Discussion

Compared with the performance of the CMP, those of the MSP1, MSP2, and MSP3 are sharply superior, mostly when the amount of error in the data increases. Indeed, the results on both the accuracy and the efficiency showed that the adaptive assessment procedure based on the CMP is more susceptible to noise than the other three.

As for the comparison among the three MSP-based procedures, a clear superiority of one of them did not emerge. Nevertheless, it can be stated that the MSP2 and MSP3 are less affected by the assumptions behind the data. In fact, they perform quite well, both in terms of accuracy and efficiency, even when the generative model was the MSPM1.

## Simulation study based on real data

The aim of this study was to test the three (MSP1, MSP2, and MSP3) adaptive procedures with real data. To this aim, a pre-existing data set (Stefanutti et al., 2021) was used that consisted of the responses of 154 subjects to the set *Q*_*g*_ of 31 ToL problems collected via a computerized version of the ToL. Among the 31 problems, only 11 were used, namely those problems belonging to the domain $Q_{g}^{(1)}$. Thus, only the goal space $\mathbf {P}_{g}^{(1)}$, and the knowledge structure $\mathcal {K}_{1}$ delineated by it, were here considered (see “Goal spaces of the Tower of London” for more details). Goal space $\mathbf {P}_{g}^{(2)}$ and the corresponding knowledge space $\mathcal {K}_{2}$ were not considered in this study because the cardinality of $\mathcal {K}_{2}$ was too large (242,498) to be used with a sample of size 154 (as resulted by the previous simulation study).

### Material and Data

The description of the administration of the ToL is briefly summarized here. Only the most important features of the administration phase are described here. For details, the reader is referred to Stefanutti et al., (2021).

To each participant, the ToL problems were administered in a randomized order via the computerized version of the ToL developed by the authors. Participants were given the following instructions: (a) solving the problems with a minimum number of moves; (b) planning in advance; (c) being as fast as possible. For every problem, the computerized ToL recorded each move until the participant made an error or correctly solved the problem. A move was considered an error whenever it reached a problem state laying outside a minimum length solution path. No time restrictions were imposed for the solution of the problems. Each participant performed an initial practice trial consisting of four problems having a different goal state from the one used for the actual test.

### Methods

The procedure used for applying each of the three MSP-based adaptive procedures was the same as that used in the previous study (see “Methods”). The only difference was that the three procedures were applied to real response patterns rather than simulated patterns. Given a subject *w* in the data set, at each step *m* ∈{1,2,⋯ ,|*Q*_*g*_|} of the assessment, the knowledge states likelihood ${\mathscr{L}}^{w}_{m}$ was updated on the basis of the responses stored in the real pattern for the problem *q* selected by the procedure at that step. Then, the modal knowledge state $\hat {K}_{m}^{w}$ at step *m* of the subject *w* was estimated. After having “dichotomized” the real data set, exactly the same steps were followed with the CMP.

The performances of the three MSP-based and of the CMP procedures were compared to one another in terms of efficiency achieved at each step *m* of the assessment. The same indexes used in the previous study were computed at each step *m* of the assessment, that is the average entropy $\bar {H}(m)$ and the proportion *p*_*m*_ of subjects exceeding a termination criterion of .50.

In case of real data, the accuracy of an adaptive assessment procedure cannot be evaluated because the true state of a subject is unknown. Nevertheless, it is possible to compare the estimated modal state $\hat {K}^{w}_{m}$ obtained at each step *m* of the assessment for subject *w* with the one estimated in the last step. In practice, the average Hamming distance $\bar {D}_{m}(\hat {K}^{w}_{11},\hat {K}_{m}^{w})$ between the two estimated modal states was computed across all subjects of the sample.

### Results

**Fig. 9:**
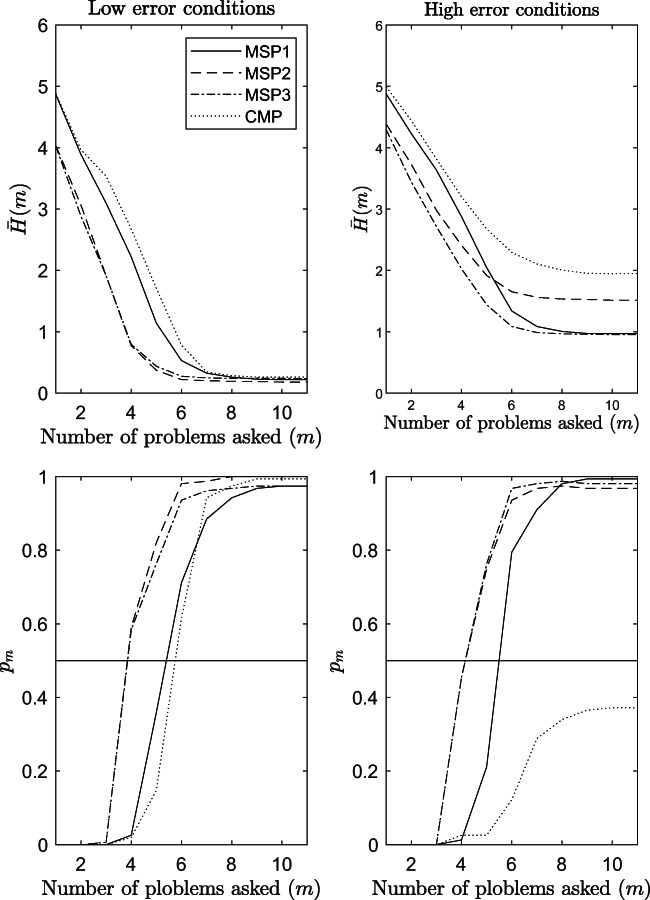
Efficiency of the adaptive procedures applied to real data. In the *upper panels*, the efficiency is given in terms of average entropy $\bar {H}_{m}$ (*y*-axis), at each step *m* of the assessment (*x*-axis). In the *lower panels*, the efficiency is given in terms of the proportion *p*_*m*_ of subjects reaching the termination criterion (*y*-axis), at each step *m* of the assessment (*x*-axis). See text for more details

Results concerning the efficiency of the procedures are depicted in Fig. 9. The upper panels of Fig. 9 show the trend of the entropy $\bar {H}(m)$ as the number of questions asked increases.

The results are quite similar to those obtained in the simulation study when the generative models were the MSPM3 (bottom panels of Fig. 8) or the MSPM2 (middle panels of Fig. 8). Indeed, with low error in the data, MSP2 and MSP3 perform in a very similar way, reaching the smallest entropy (about 0) with the smallest number of problems asked (six problems out of 11). With a higher amount of error in the data, the best performances are obtained by the MSP3 and the MSP1, whereas the MSP2 and the CMP obtained worse performances. In particular, the entropy reached by the CMP is two times worse than that of the MSP3 and MSP1.

The lower panels of Fig. 9 show the trend of the proportion *p*_*m*_ of subjects reaching the termination criterion as the number of questions asked increases. Even for this performance index about the efficiency of the procedures, similar results as those obtained in the simulation study can be drawn. With low error in the data, almost the whole sample reaches the termination criterion in five questions with the MSP2 and MSP3. For the other two procedures, the same result is obtained with at least two questions more. With the high amount of error in the data, the performance of the CMP drastically reduces. Nevertheless, it is interesting to note that in these conditions, the performance of the CMP reached by using real data is about twice the one obtained by the same model in the simulation study (see Fig. 7, bottom right panel). This last results could suggest that the amount of noise in the data set could be in between the “low error” and “high error” conditions examined in the simulation study illustrated in “Simulation study”.

## General discussion

In the present research, three adaptive procedures for the assessment of procedural skills have been proposed. These procedures are based on the Markov solution processes model (Stefanutti et al., 2021), and they use the sequence of moves observed in the solution of a problem to increase the assessment efficiency and accuracy. The three adaptive assessment procedures differ from one another in the assumption underlying the solution process. The pre-planning assumption states that the solution to the problem is entirely planned before the first move. According to the interim-planning assumption, planning can occur during the execution of the problem. Finally, the mixed-planning assumption allows both pre-planning and interim planning.

The aforementioned assessment procedures were implemented in MATLAB and they were used for running two simulation studies. In the first simulation study, the data sets were generated under the three different assumptions with the aim of comparing the capability of the three procedures to recover the true knowledge state of the individual. The performances of three procedures were compared to one another and with that of a baseline procedure represented by the CMP (Falmagne and Doignon, 1988). Results showed that all of them outperformed the CMP. Regarding the accuracy, the performance of the CMP was as good as that of the MSP1-based procedure (and better than the other two) only in the conditions in which the generative model was the MSPM1, and the amount of error was low. In all the other conditions, the MSP-based procedures outperform the CMP. Regarding the efficiency, the MSP2- and MSP3-based procedures performed better than the other two in almost all the conditions.

In the second simulation study, the procedures were applied to a real data set of 154 individuals to whom a set of the ToL problems was administered. The results were coherent with those obtained in the first simulation study. An exception is the case of the condition of high error, where for the MSP1- and MSP3-based procedures the entropy of the knowledge states likelihood distribution was almost the same and the lowest. This may seem as an incoherence with the first simulation study. A tentative explanation is that the participants were instructed to plan in advance the whole solution paths. However, some participants could have applied a different strategy.

The main peculiarity of the procedures presented in this article is that the dependencies among problems reflect their structural relations in the problem space rather than inferred through the application of statistical procedures to the data. Such a relationship is based on the assumption that, if a solution path includes another solution path, then an individual who knows how to apply the former also knows how to apply the latter. Referring to Example 1 in “Adaptive assessment in a problem space”, an individual who knows how to solve problem *s*_1_ by applying $ab\bar {a}\bar {b}\bar {a}$ will also be able to solve *s*_3_ by applying $b\bar {a}\bar {b}\bar {a}$. The validity of this assumption seems reasonable, although it needs to be empirically tested in every single context where such procedures are applied. For instance, in the context of the ToL test, empirical validation of the MSPM by (Stefanutti et al., 2021) showed promising results.

The outcome of a PKST-based assessment procedure is a knowledge state rather than a numerical score. The knowledge state is an “estimated” representation of the portion of the problem space that is known to the problem solver, or the portion where this last can operate successfully. This kind of representation cannot by achieved through a simple numerical score. This seems to be a clear advantage of the proposed approach, in the attempt of better capturing and explaining individual differences.

In the clinical context, many advantages of this representation may be pointed out. In KST, a knowledge state has two well-known properties that are named the “inner fringe” and the “outer fringe”. Both of these have very clear and theoretically well-founded interpretation in the educational context (Falmagne et al., 2013). The inner fringe represents the points of strength of the student, whereas the outer fringe represents what a student is ready to learn. Such interpretations can be easily transferred to the clinical and psychological contexts. The inner fringe represents the maximum performance of the individual, which is not the same thing as number of problems solved correctly. The outer fringe contains the problems that are one step ahead for the individual. In a rehabilitation context, they may be used as training exercises, which are at the appropriate difficulty level for the patient.

This work was focused on comparing the updating rule of CMP and MSP-based procedures. However, other aspects of the assessment procedure can be varied to increase the efficiency of an assessment. For instance, (Heller & Repitsch, 2012) has shown that using an informative initial likelihood distribution on the knowledge states can improve the performance of an assessment procedure. However, an incorrect initial distribution can impair the performance of the procedure. In this application, the uniform distribution was used to avoid those issues. However, future applications should further investigate these aspects to further improve the assessment performance.

From a practical perspective, a field of application for the adaptive procedures proposed in this research is neuropsychological testing. In the last years, the attention of neuropsychology researchers has focused on how modern psychometric theories and advances in technology should be incorporated in neuropsychological assessment (see, e.g., Costa, Dogan, Schulz, & Reetz, 2019; Howieson, 2019; Kessels, 2019; Marcopulos & Łojek, 2019). Some attempts and innovations were made, such as a recent work by D’Alessandro et al., (2020) which used a computational model approach to assess perseverant behavior with healthy and substance-dependent individuals on the Wisconsin Card Sorting Task. Although based on a different approach, the assessment procedures proposed in this article have a similar objective.

Another promising field of application is serious games. The procedures developed in this article can be used as a base for the definition of educational games and virtual training environments. This sets up an agenda for future research work.

## Open Practices Statement

The code and an example of how to run it on an existing simulated dataset are available at the following link: https://osf.io/qa8mg/?view_only=8b4e148300de40a6941df4a102067fc1/. None of the experiments was preregistered.

### Electronic supplementary material

Below is the link to the electronic supplementary material.

(ZIP 1.37 MB)

## References

[CR1] Berg WK, Byrd DL (2002). The Tower of London spatial problem-solving task: Enhancing clinical and research implementation. Journal of Clinical and Experimental Neuropsychology.

[CR2] Berg WK, Byrd DL, McNamara JPH, Case K (2010). Deconstructing the tower: Parameters and predictors of problem difficulty on the Tower of London task. Brain and Cognition.

[CR3] Bolt D (2007). The present and future of IRT-based cognitive diagnostic models (ICDMs) and related methods. Journal of Educational Measurement.

[CR4] Costa AS, Dogan I, Schulz JB, Reetz K (2019). Going beyond the mean: Intraindividual variability of cognitive performance in prodromal and early neurodegenerative disorders. The Clinical Neuropsychologist.

[CR5] D’Alessandro M, Radev ST, Voss A, Lombardi L (2020). A Bayesian brain model of adaptive behavior: An application to the Wisconsin card sorting task. PeerJ.

[CR6] de Chiusole D, Stefanutti L, Anselmi P, Robusto E (2020). Stat-knowlab. Assessment and learning of statistics with competence-based knowledge space theory. International Journal of Artificial Intelligence in Education.

[CR7] de la Torre J (2009). DINA model and parameter estimation: A didactic. Journal of Educational and Behavioral Statistics.

[CR8] DiBello LV, Stout W (2007). Guest editors’ introduction and overview: IRT-based cognitive diagnostic models and related methods. Journal of Educational Measurement.

[CR9] Doignon J-P (1994). Knowledge spaces and skill assignments.

[CR10] Doignon J-P, Falmagne J-C (1985). Spaces for the assessment of knowledge. International Journal of Man-Machine Studies.

[CR11] Doignon J-P, Falmagne J-C (1999). Knowledge spaces.

[CR12] Donadello I, Spoto A, Sambo F, Badaloni S, Granziol U, Vidotto G (2017). ATS-PD: An adaptive testing system for psychological disorders. Educational and Psychological Measurement.

[CR13] Düntsch I, Gediga G (1995). Skills and knowledge structures. British Journal of Mathematical and Statistical Psychology.

[CR14] Falmagne J-C, Doignon J-P (1988). A class of stochastic procedures for the assessment of knowledge. British Journal of Mathematical and Statistical Psychology.

[CR15] Falmagne J-C, Doignon J-P (2011). Learning spaces.

[CR16] Falmagne J-C, Koppen M, Villano M, Doignon J-P, Johanessen L (1990). Introduction to knowledge spaces: How to build, test and search them. Psychological Review.

[CR17] Falmagne, J-C, Albert, D., Doble, C., Eppstein, D., & Hu, X. (2013). Knowledge spaces: Applications in education. Springer Science & Business Media.

[CR18] Funke J (2013). Human problem solving in 2012. The Journal of Problem Solving.

[CR19] Gediga G, Düntsch I (2002). Skill set analysis in knowledge structures. British Journal of Mathematical and Statistical Psychology.

[CR20] Granziol, U., Brancaccio, A., Pizziconi, G., Spangaro, M., Gentili, F., Bosia, M., ..., et al. (2020). On the implementation of computerized adaptive observations for psychological assessment. Assessment, 1073191120960215. 10.1177/1073191120960215 33016093

[CR21] Heller J, Repitsch C (2012). Exploiting prior information in stochastic knowledge assessment. Methodology.

[CR22] Heller, J., Augustin, T., Hockemeyer, C., Stefanutti, L., & Albert, D. (2013). Recent developments in competence-based knowledge space theory. In *Knowledge spaces* (pp. 243–286): Springer.

[CR23] Heller J, Stefanutti L, Anselmi P, Robusto E (2015). On the link between cognitive diagnostic models and knowledge space theory. Psychometrika.

[CR24] Heller, J., Ünlü, A, & Albert, D. (2013). Skills, competencies and knowledge structures. In *Knowledge spaces* (pp. 229–242): Springer.

[CR25] Howieson D (2019). Current limitations of neuropsychological tests and assessment procedures. The Clinical Neuropsychologist.

[CR26] Jonassen DH (2000). Toward a design theory of problem solving. Educational Technology Research and Development.

[CR27] Kaller CP, Rahm B, Köstering L, Unterrainer JM (2011). Reviewing the impact of problem structure on planning: A software tool for analyzing tower tasks. Behavioural Brain Research.

[CR28] Kaller CP, Unterrainer JM, Rahm B, Halsband U (2004). The impact of problem structure on planning: Insights from the Tower of London task. Cognitive Brain Research.

[CR29] Kessels RPC (2019). Improving precision in neuropsychological assessment: Bridging the gap between classic paper-and-pencil tests and paradigms from cognitive neuroscience. The Clinical Neuropsychologist.

[CR30] Korossy K (1997). Extending the theory of knowledge spaces: A competence-performance approach. Zeitschrift für Psychologie.

[CR31] Korossy, K. (1999). Modeling knowledge as competence and performance. In D Albert, & J Lukas (Eds.) *Knowledge Spaces: Theories, Empirical Research, Applications* (pp. 103–132). Mahwah, NJ: Lawrence Erlbaum Associates.

[CR32] Langley, P., Magnani, L., Schunn, C., & Thagard, P. (2005). An extended theory of human problem solving. In *Proceedings of the Annual Meeting of the Cognitive Science Society*, Vol. 27.

[CR33] Marcopulos B, Łojek E (2019). Introduction to the special issue: Are modern neuropsychological assessment methods really “modern”? reflections on the current neuropsychological test armamentarium. The Clinical Neuropsychologist.

[CR34] McKinlay A, Kaller CP, Grace RC, Dalrymple-Alford JC, Anderson TJ, Fink J, Roger D (2008). Planning in Parkinson’s disease: A matter of problem structure?. Neuropsychologia.

[CR35] Newell, A., & Simon, H. A. (1972). Human problem solving. (Vol. 104) (No. 9). Prentice Hall Englewood Cliffs, NJ.

[CR36] Newman SD, Pittman G (2007). The Tower of London: A study of the effect of problem structure on planning. Journal of Clinical and Experimental Neuropsychology.

[CR37] Shallice T (1982). Specific impairments of planning. Philosophical Transactions of the Royal Society of London. B, Biological Sciences.

[CR38] Shannon CE (1948). A mathematical theory of communication. The Bell System Technical Journal.

[CR39] Stefanutti L (2019). On the assessment of procedural knowledge: From problem spaces to knowledge spaces. British Journal of Mathematical and Statistical Psychology.

[CR40] Stefanutti L, Albert D (2003). Skill assessment in problem solving and simulated learning environments. J. UCS.

[CR41] Stefanutti L, de Chiusole D (2017). On the assessment of learning in competence based knowledge space theory. Journal of Mathematical Psychology.

[CR42] Stefanutti L, de Chiusole D, Brancaccio A (2021). Markov solution processes: Modeling human problem solving with procedural knowledge space theory. Journal of Mathematical Psychology.

[CR43] Tatsuoka, K. (1990). Toward an integration of item-response theory and cognitive error diagnosis. In *Monitoring skills and knowledge acquisition* (pp. 453–488): Hillsdale: Lawrence Erlbaum Associates.

[CR44] Ünlü, A, Schrepp, M., Heller, J., Hockemeyer, C., Wesiak, G., & Albert, D. (2013). Recent developments in performance-based knowledge space theory. In *Knowledge Spaces* (pp. 147–192): Springer.

[CR45] Zhang J, Norman DA (1994). Representations in distributed cognitive tasks. Cognitive Science.

