# Supplementary material for: Algorithms for the adaptive assessment of procedural knowledge and skills
Source: Behav Res Methods. 2022 Dec 16;55(7):3929–51. doi: 10.3758/s13428-022-01998-y (PMC10616228; doi:10.3758/s13428-022-01998-y)
Supplement: Supplementary file 1 — (ZIP 1.37 MB) [file 13428_2022_1998_MOESM1_ESM.zip › SupplementaryMaterial/SuppMat.pdf]

Web Supplementary Material of the article "Algorithms for the adaptive assessment of procedural knowledge and skills"

A list of figures follows. Each figure displays the results obtained in a different condition of the simulation study (see Section 4.1 of the article for a detailed description of the simulation design).

Figures 1 and 2 show, respectively, the accuracy and efficiency performances of the algorithms when the knowledge structure was $\mathcal{K}_1$ and the sample size was 1,000 (even conditions 2 to 12).

Figures 3 to 7 show the results of simulation conditions 13 to 24, when the knowledge structure was $\mathcal{K}_2$ and the sample sizes were 1,000 or 100,000. In particular Figures 3 and 5 refer to the accuracy performances of the algorithms, whereas Figure 4, 6, and 7 refer to the efficiency performances.

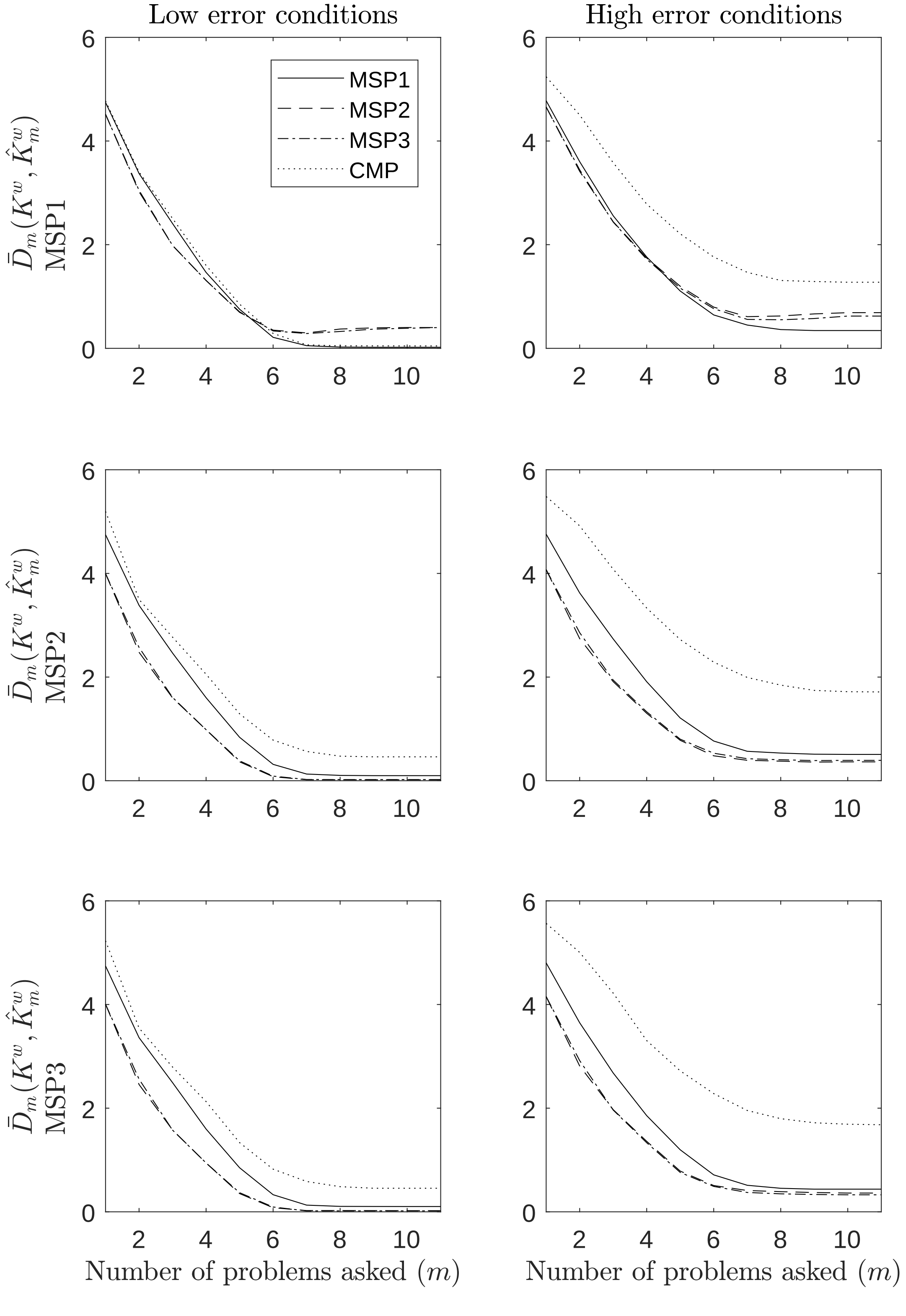

Figure 1: Accuracy of the algorithms in terms of average Hamming distance between the true and the estimated knowledge state in even conditions 2 to 12, when the sample size was 1,000 and the knowledge structure was $\mathcal{K}_1$.

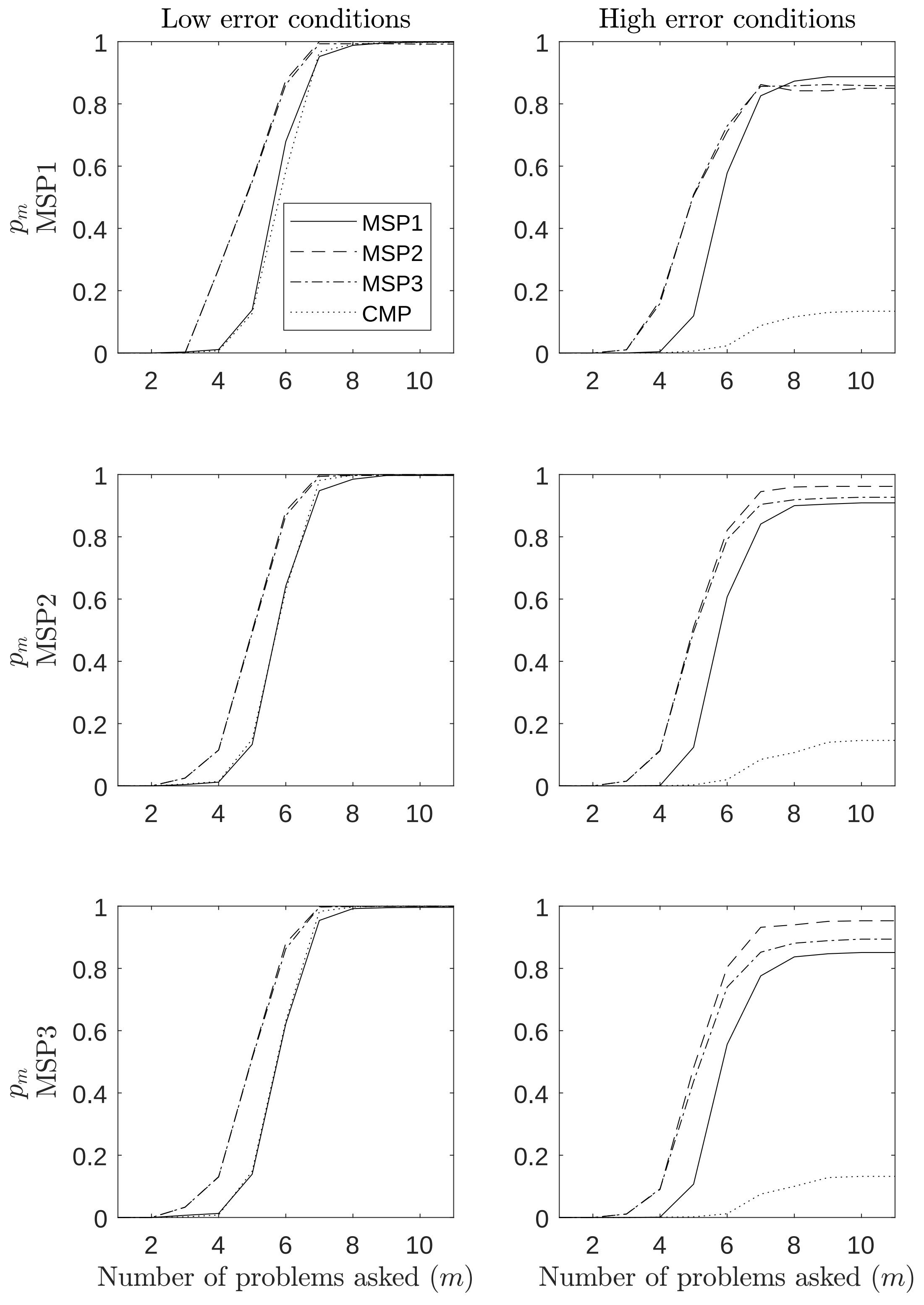

Figure 2: Efficiency of the algorithms in terms of proportion of subjects that reached the termination criterion $p \geq .50$ at step m. The results refers to even conditions 2 to 12 of the simulation study.

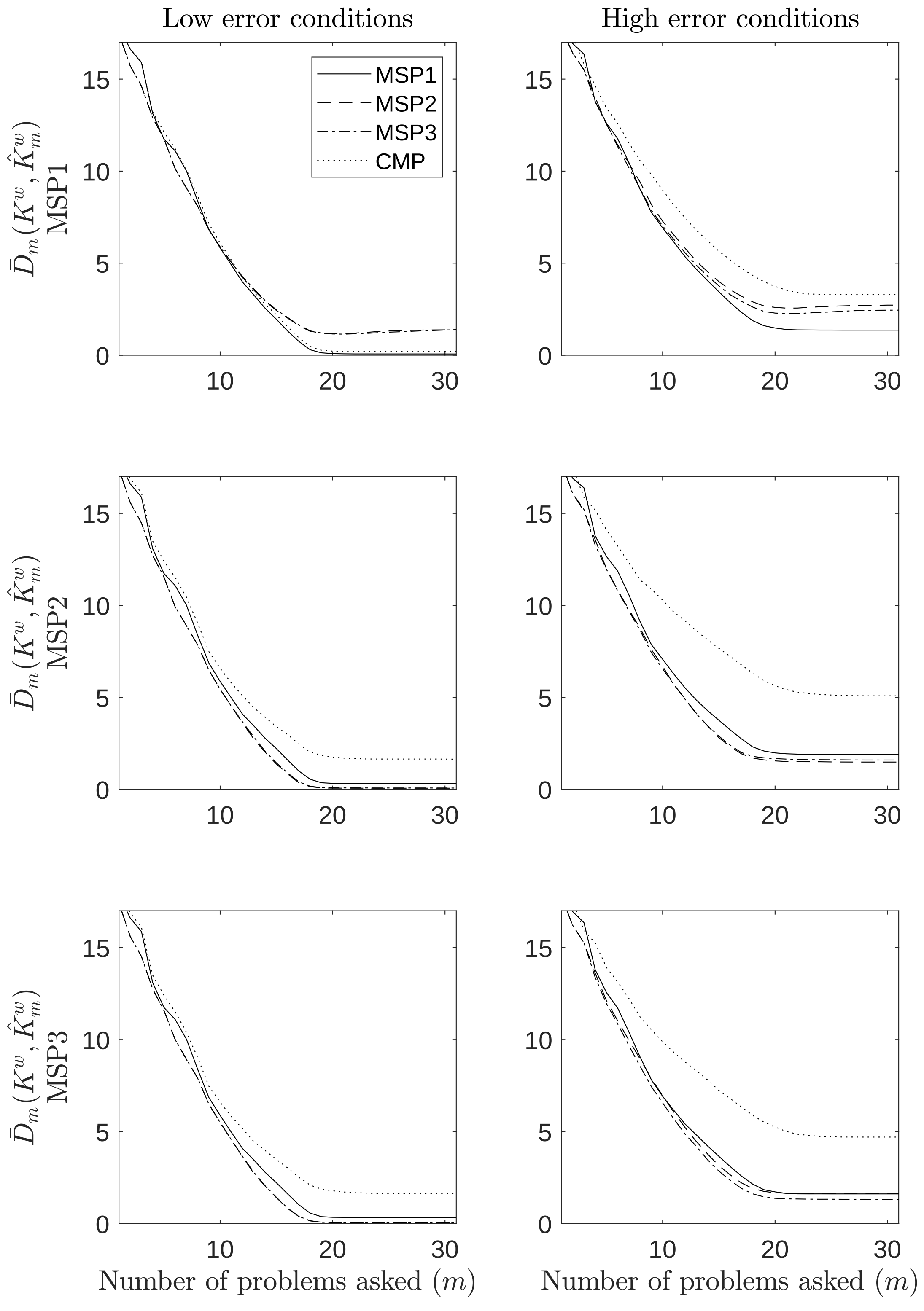

Figure 3: Accuracy of the algorithms in terms of average Hamming distance between the true and the estimated knowledge state in odds conditions 12 to 24, when the sample size was 1,000 and the knowledge structure was $\mathcal{K}_2$.

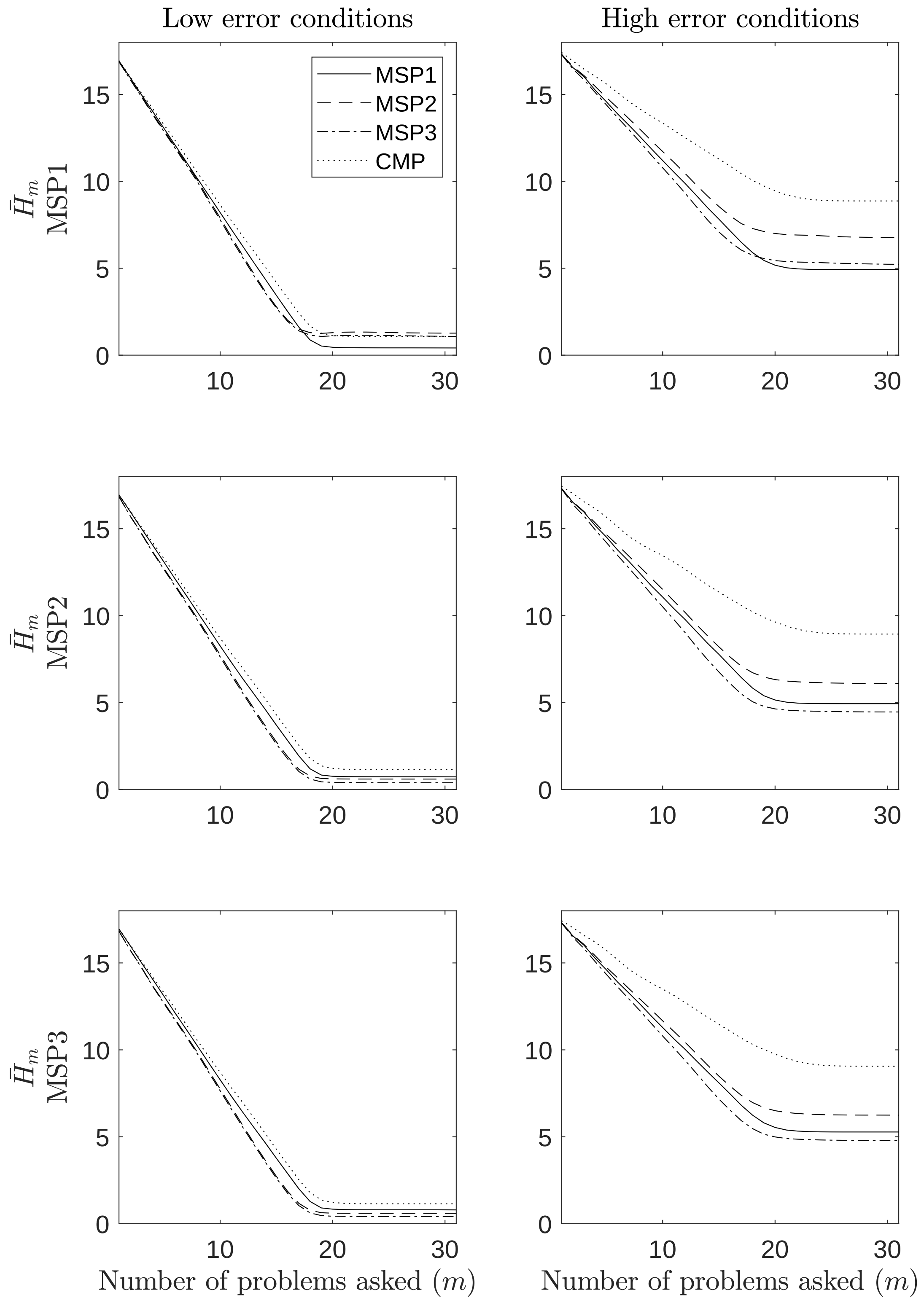

Figure 4: Efficiency of the adaptive algorithms in terms of average entropy $\bar{H}_m$ at each step m of the assessment in odds conditions 12 to 24 of the simulation study (N =100,000 and knowledge structure $\mathcal{K}_2$).

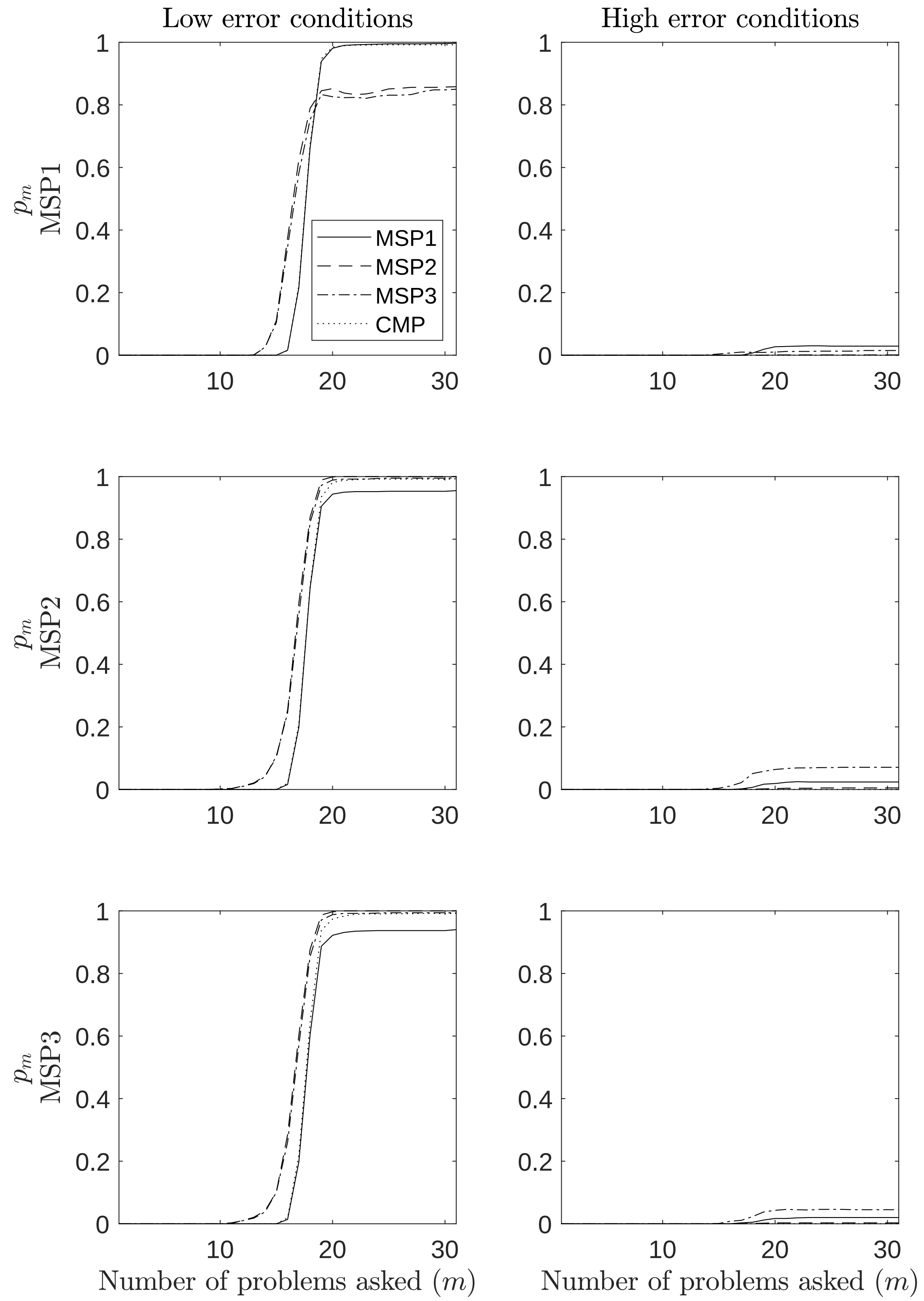

Figure 5: Efficiency of the algorithms in terms of proportion of subjects that reached the termination criterion $p \geq .50$ at step m. The results refers to odds conditions 12 to 24, when the sample size was 1,000 and the knowledge structure was $\mathcal{K}_2$.

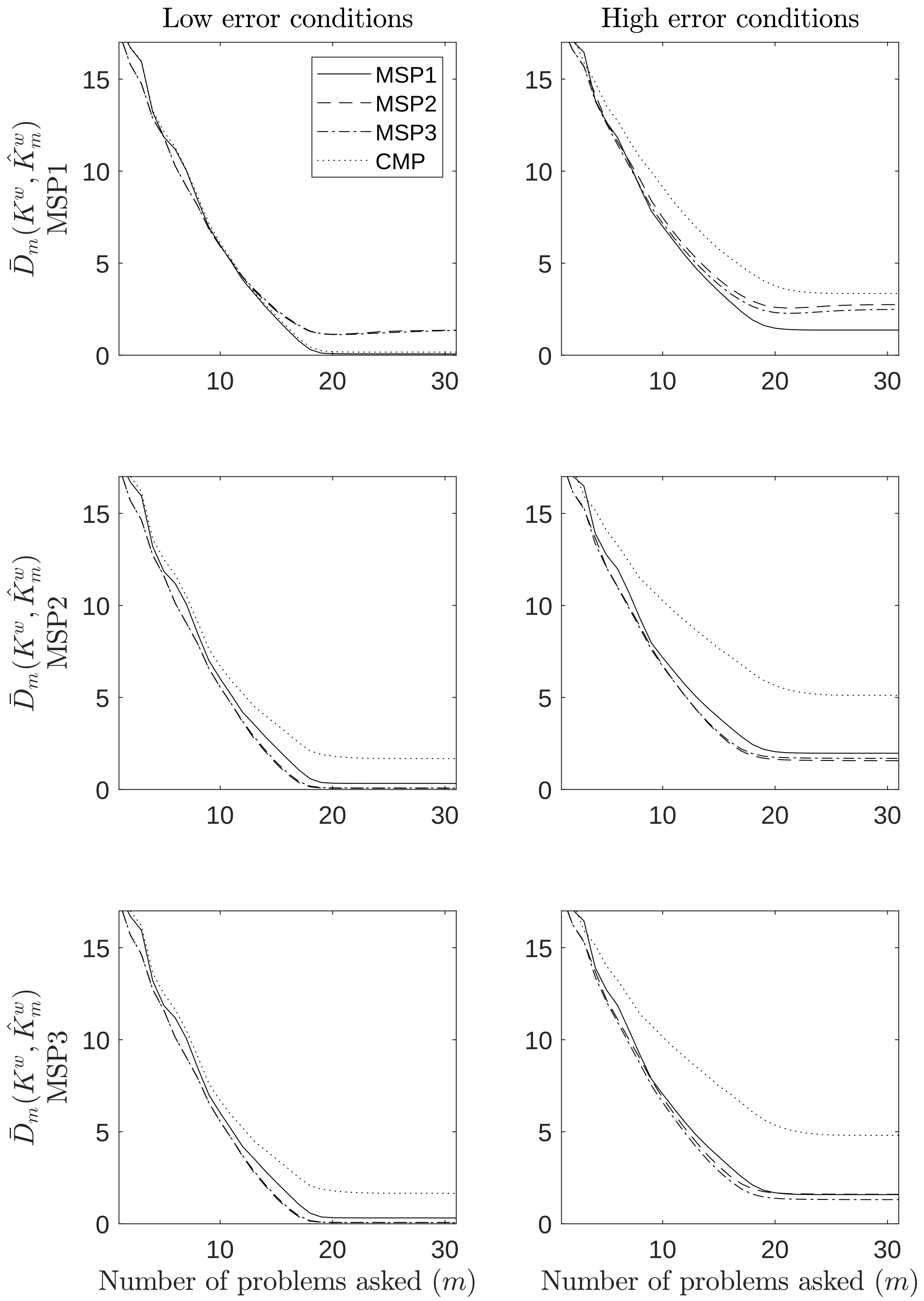

Figure 6: Accuracy of the algorithms in terms of average Hamming distance between the true and the estimated knowledge state in even conditions 13 to 23, when the sample size was 100,000 and the knowledge structure was $\mathcal{K}_2$.

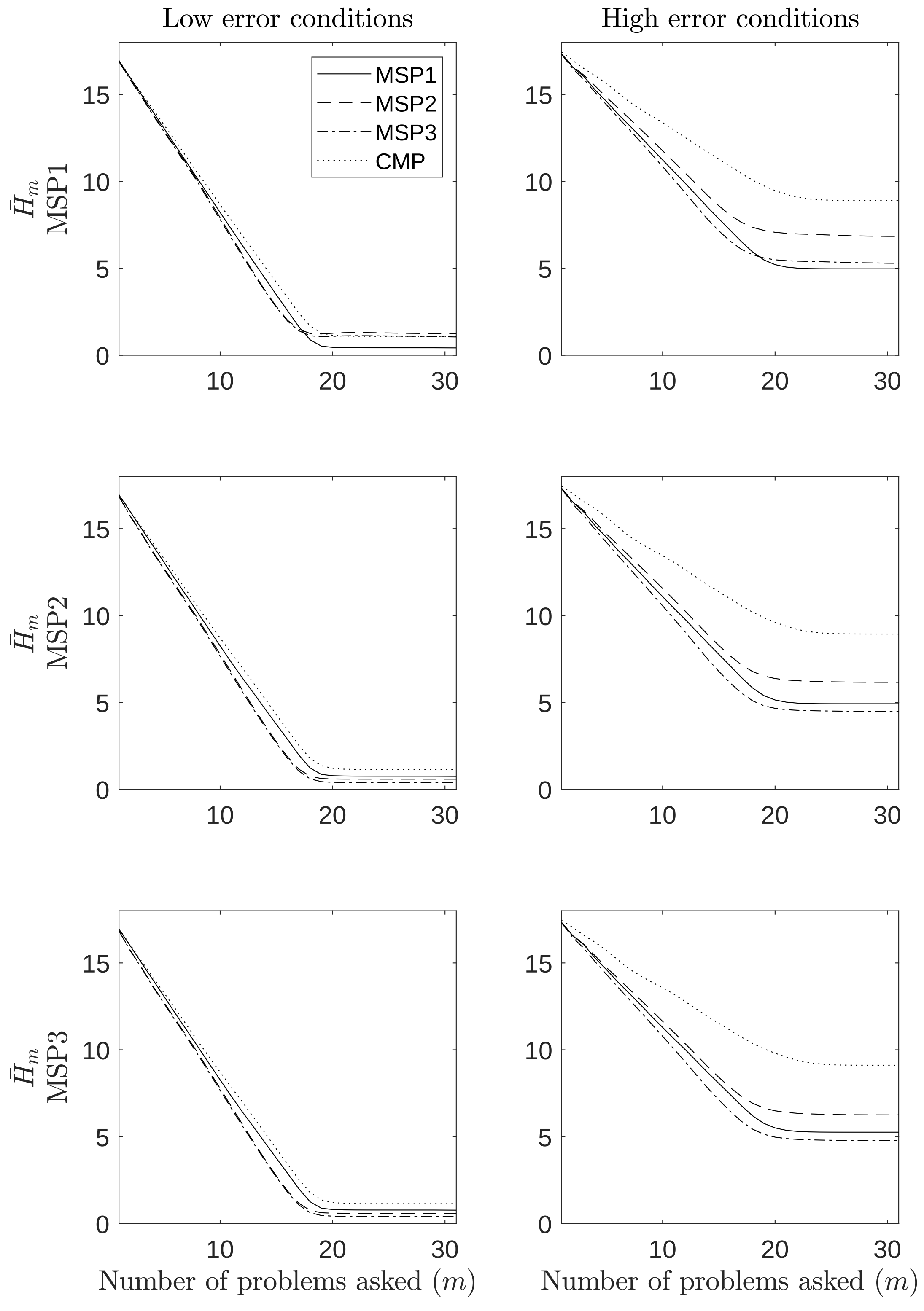

Figure 7: Efficiency of the adaptive algorithms in terms of average entropy $\bar{H}_m$ at each step m of the assessment in even conditions 13 to 23 of the simulation study (N =100,000 and knowledge structure $\mathcal{K}_2$).

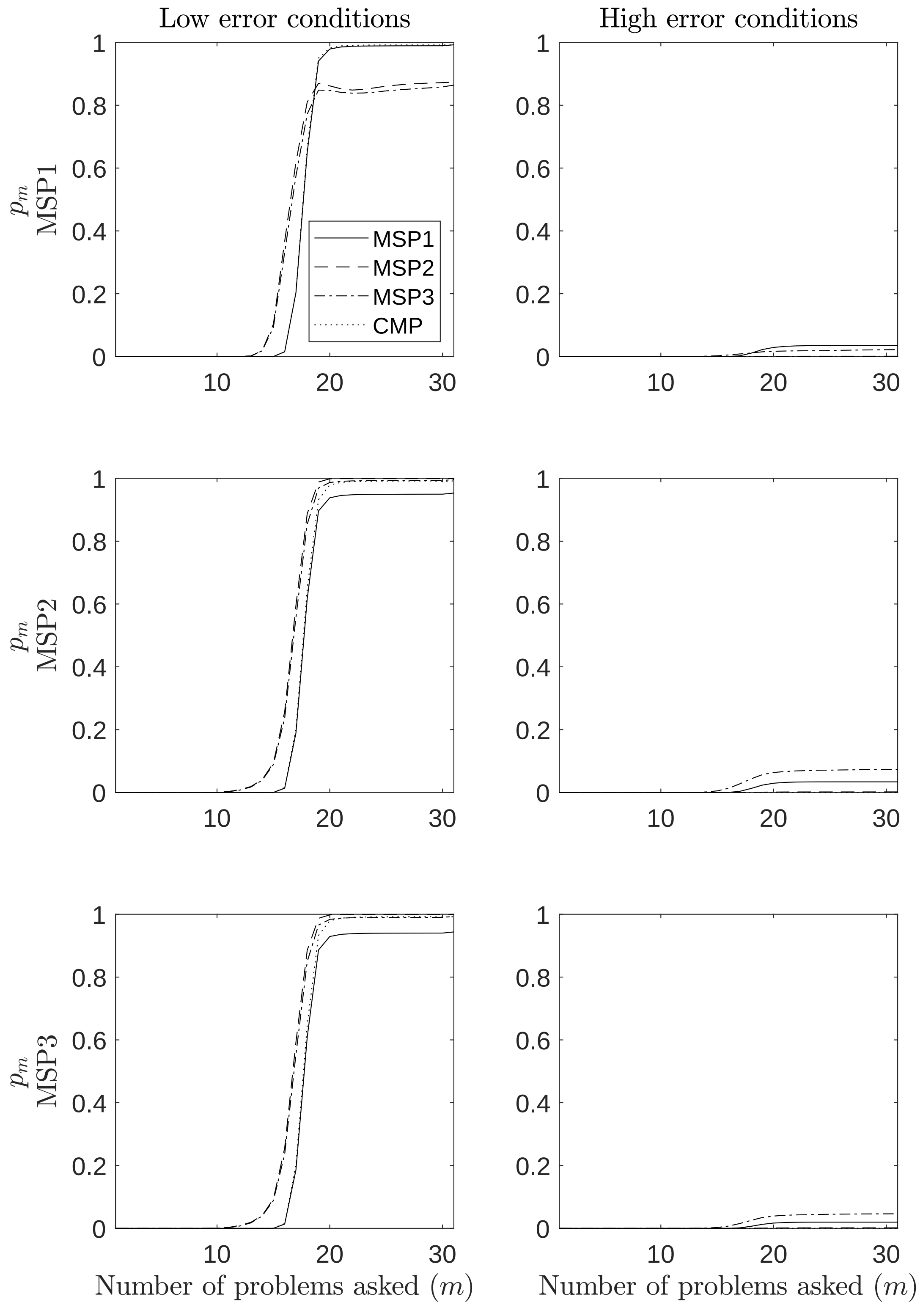

Figure 8: Efficiency of the algorithms in terms of proportion of subjects that reached the termination criterion $p \geq .50$ at step m. The results refers to even conditions 13 to 23, when the sample size was 100,000 and the knowledge structure was $\mathcal{K}_2$.
