# Supplementary figures and images for: Algorithms for the adaptive assessment of procedural knowledge and skills
Source: Behav Res Methods. 2022 Dec 16;55(7):3929–51. doi: 10.3758/s13428-022-01998-y (PMC10616228; doi:10.3758/s13428-022-01998-y)

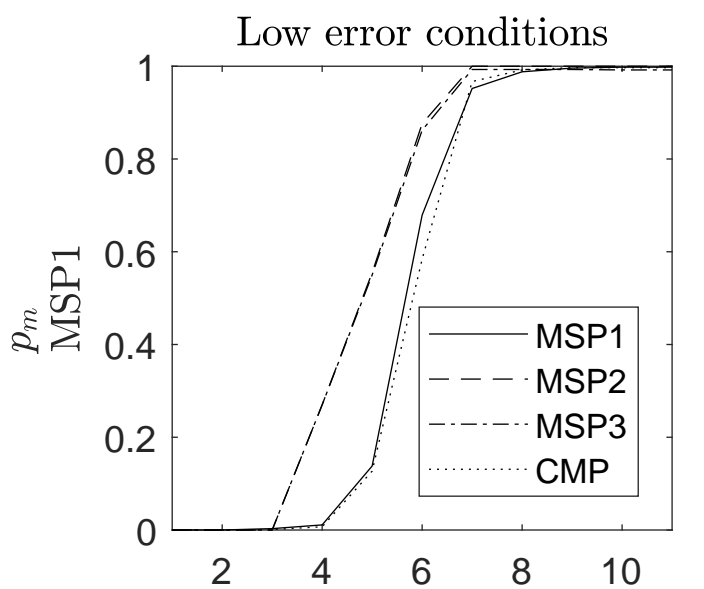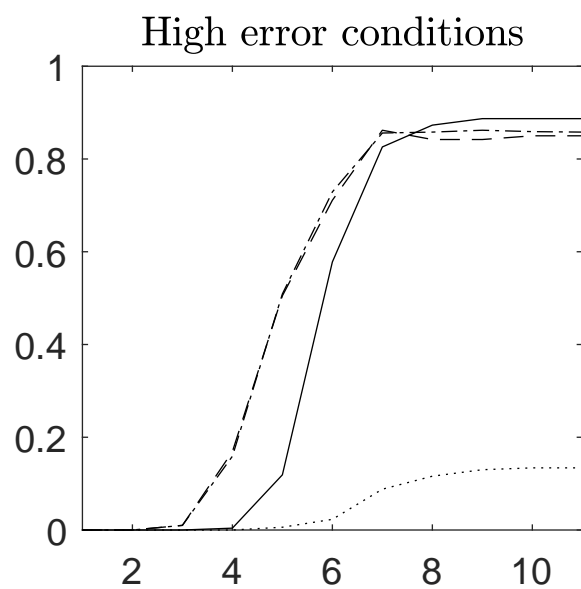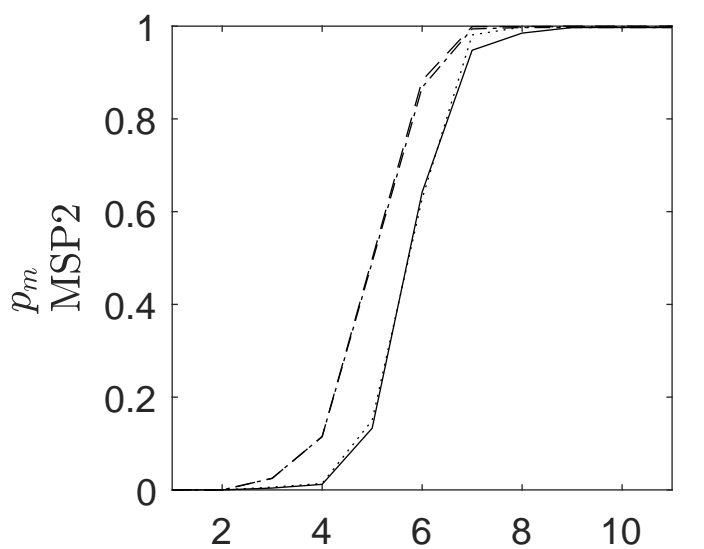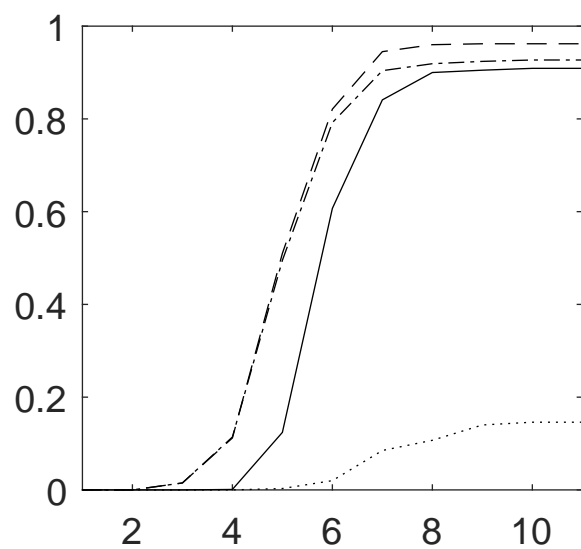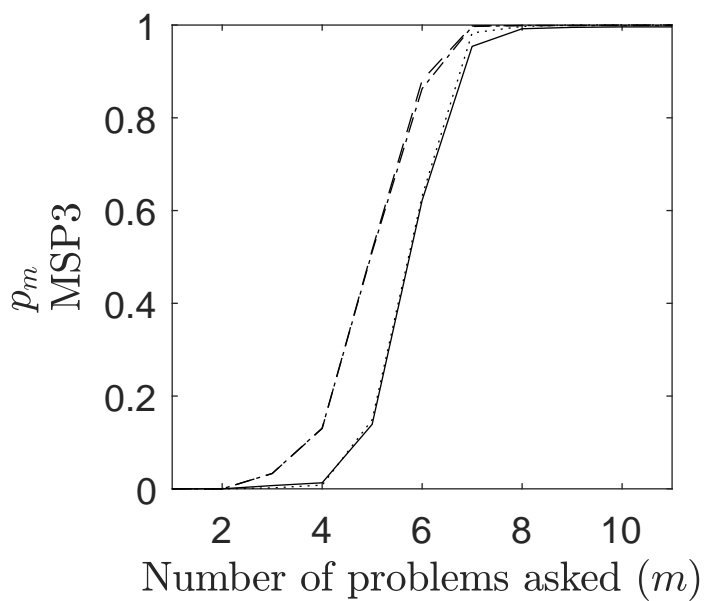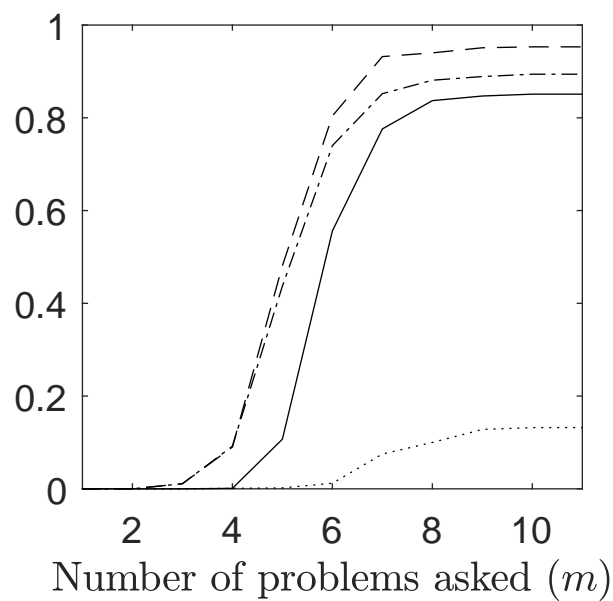

Supplement: Supplementary file 1 — (ZIP 1.37 MB) [file 13428_2022_1998_MOESM1_ESM.zip › SupplementaryMaterial/efficiency_numbesubject_1000_K1.pdf]

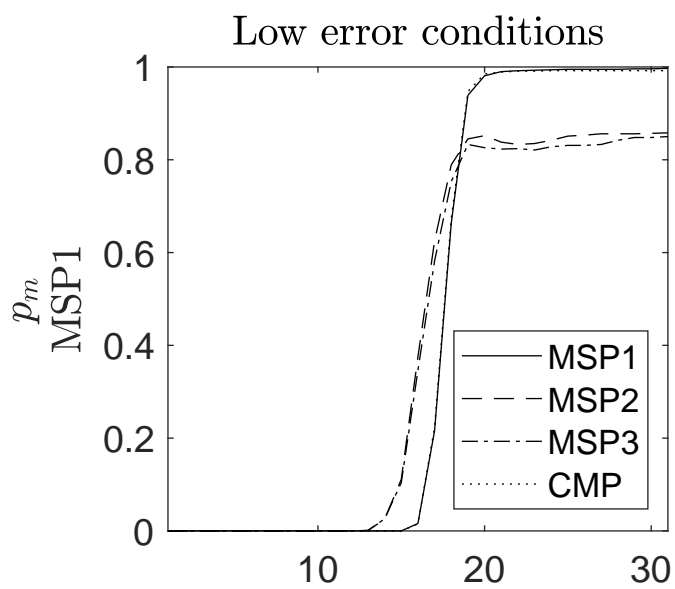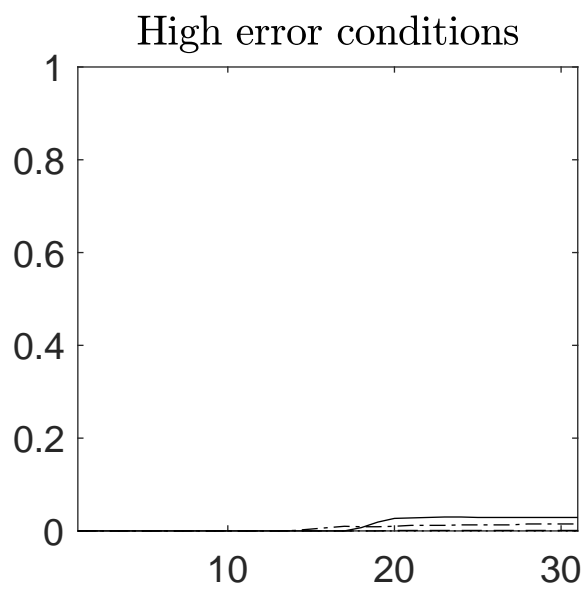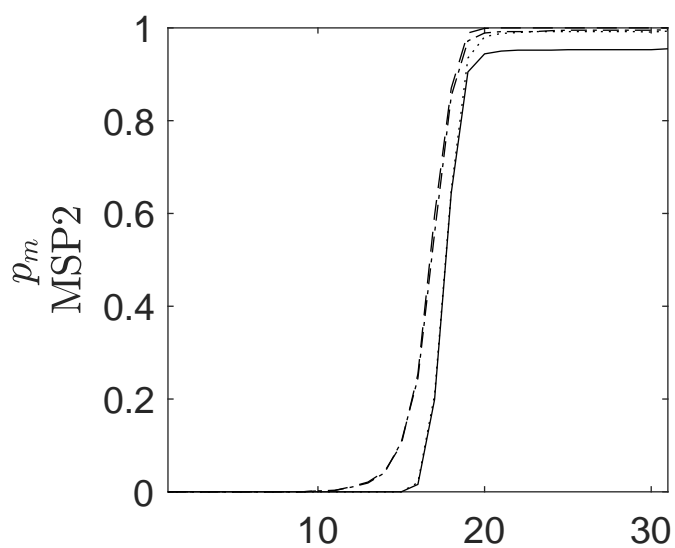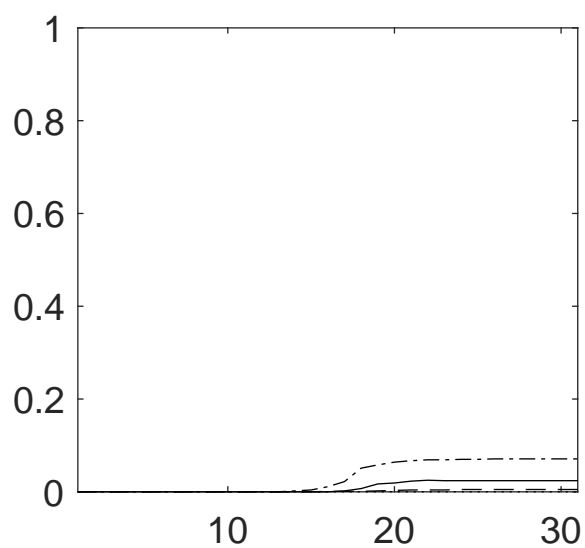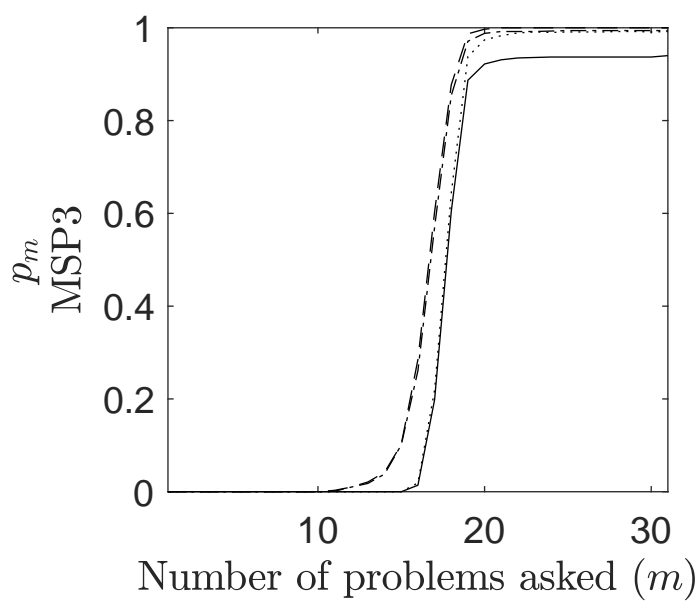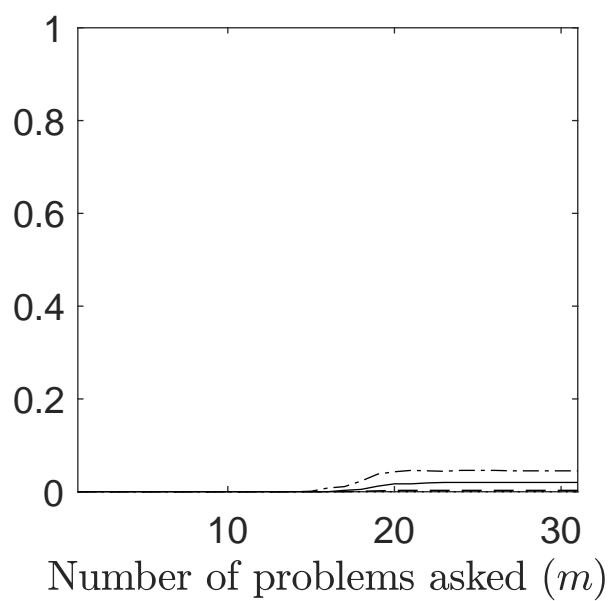

Supplement: Supplementary file 1 — (ZIP 1.37 MB) [file 13428_2022_1998_MOESM1_ESM.zip › SupplementaryMaterial/efficiency_numbesubject_1000_K2.pdf]

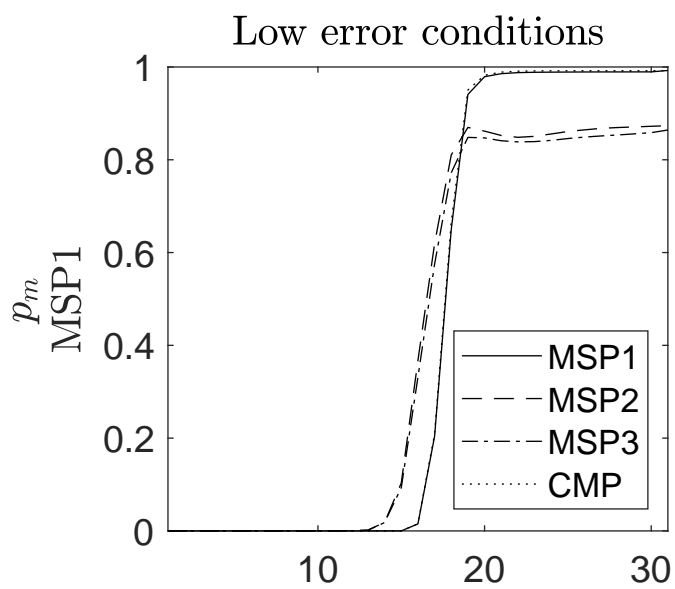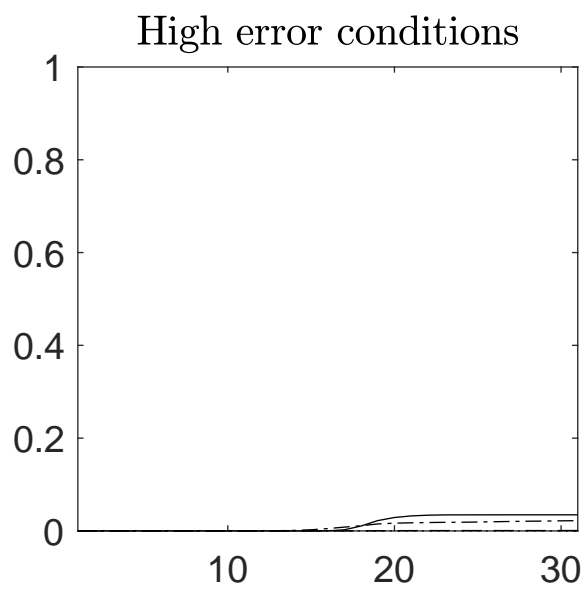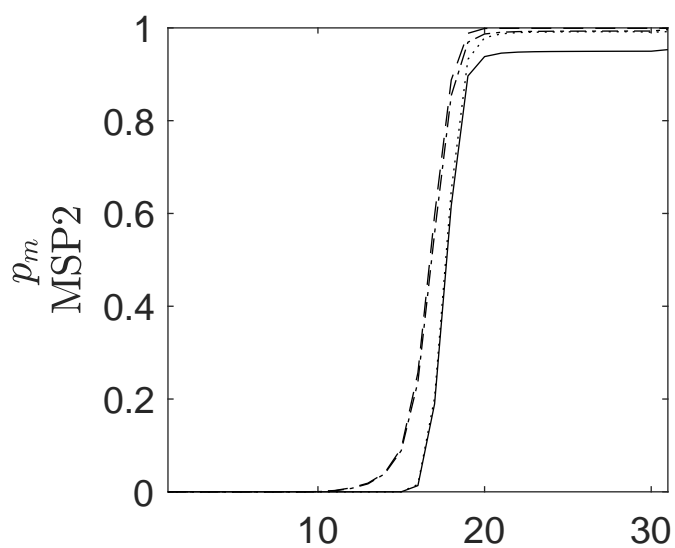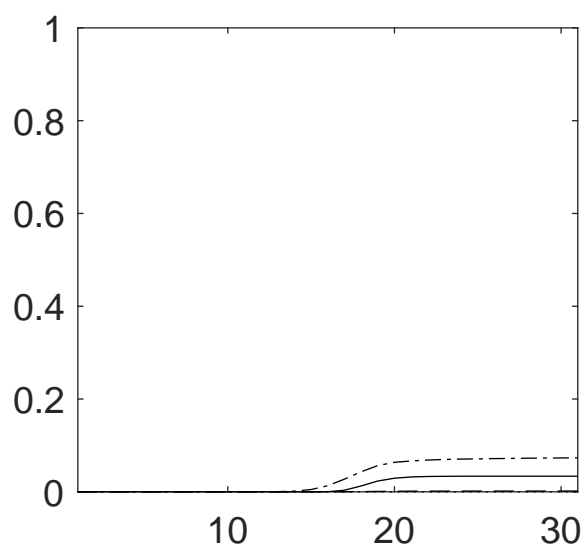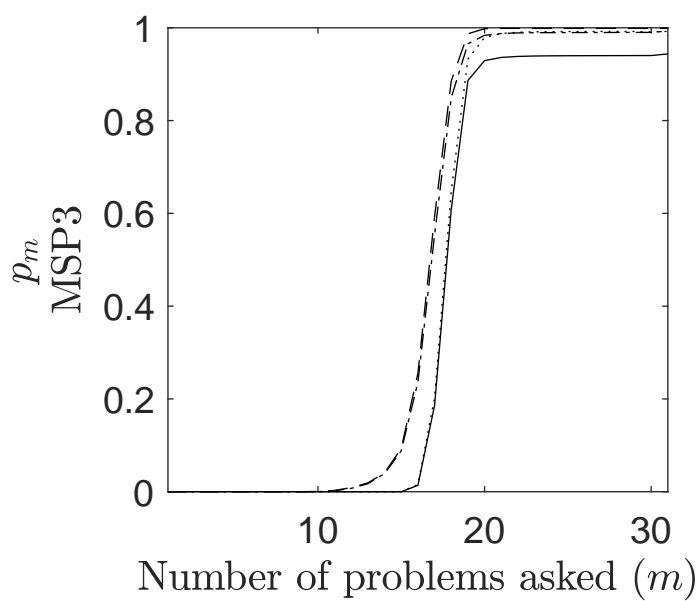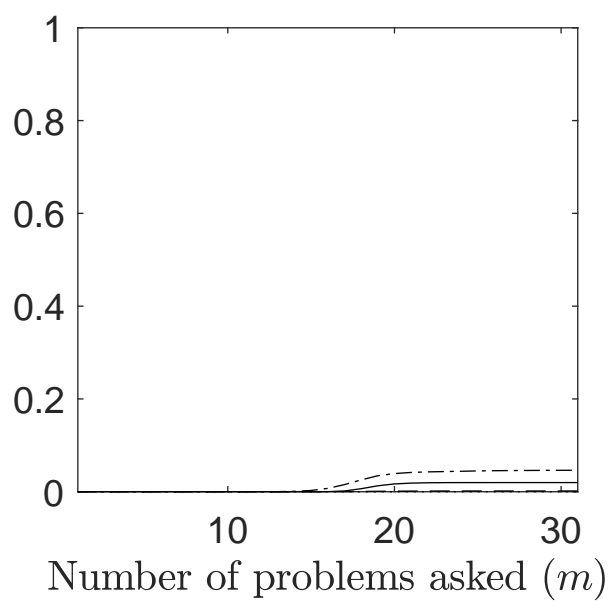

Supplement: Supplementary file 1 — (ZIP 1.37 MB) [file 13428_2022_1998_MOESM1_ESM.zip › SupplementaryMaterial/efficiency_numbesubject_100K_K2.pdf]

Low error conditions

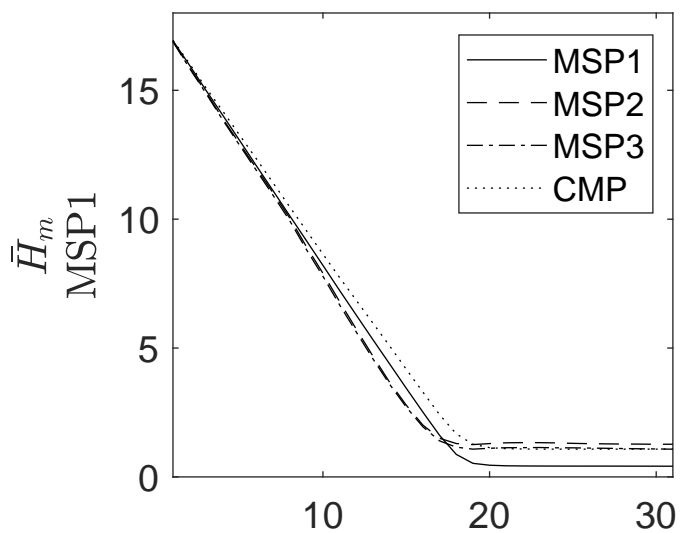

High error conditions

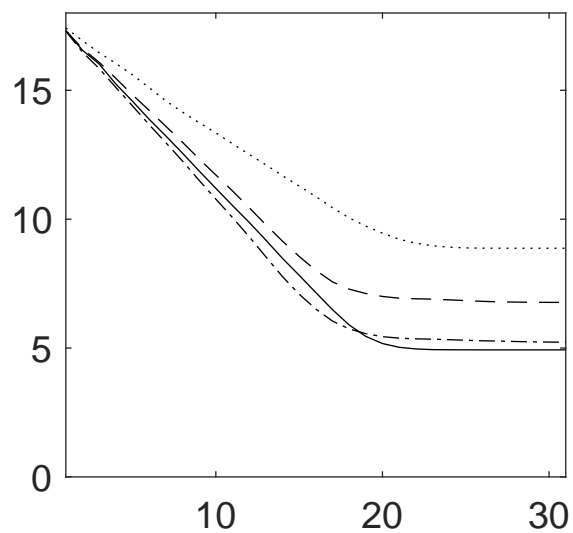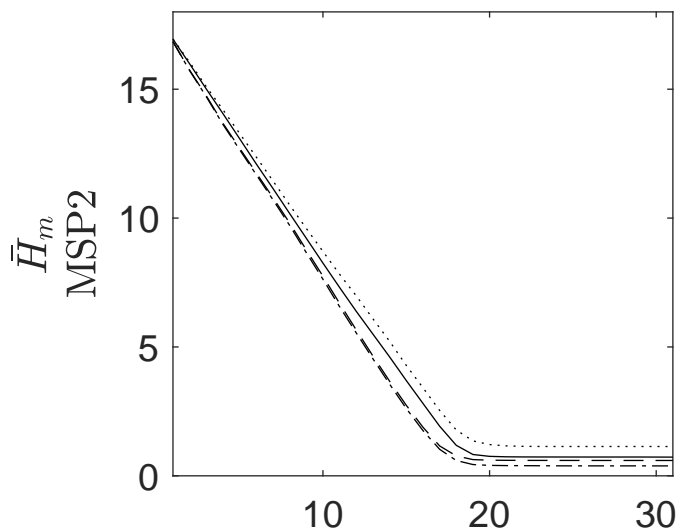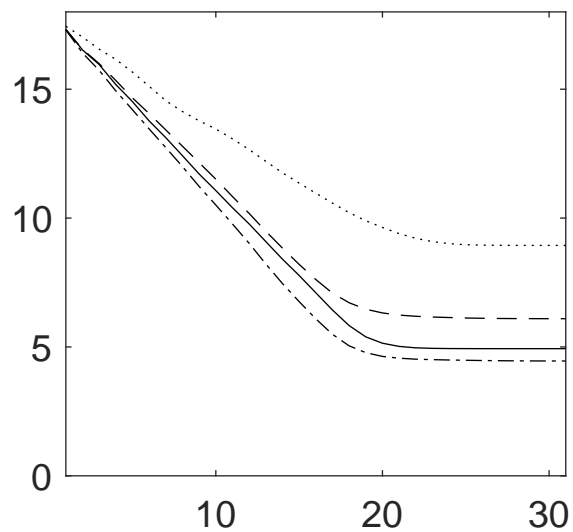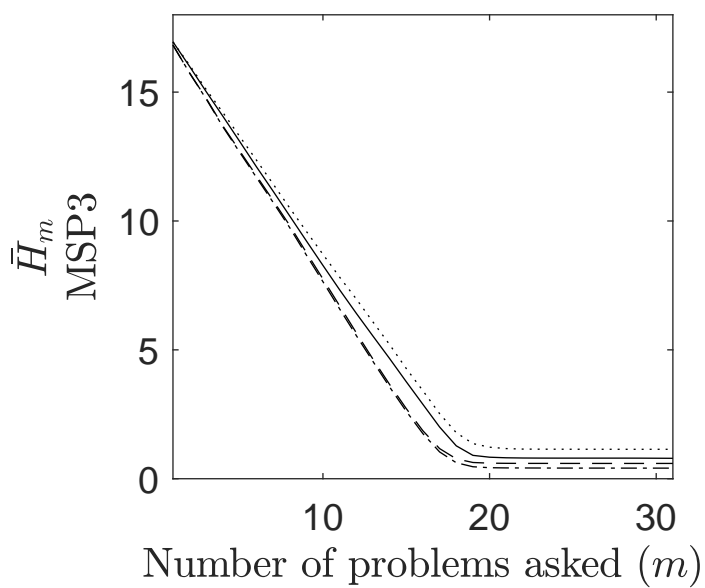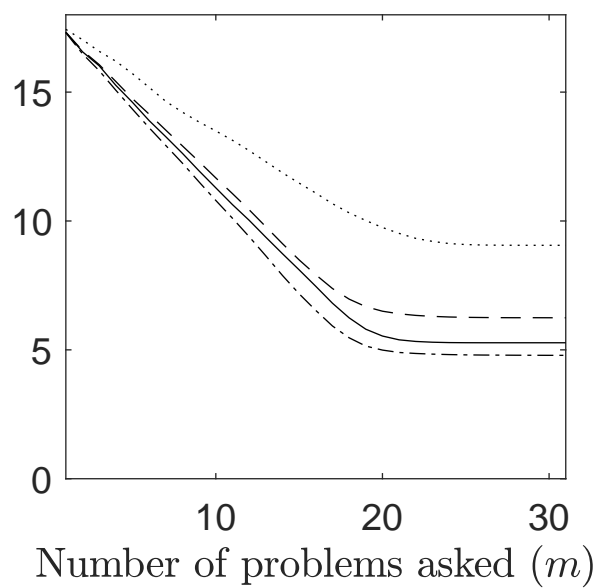

Supplement: Supplementary file 1 — (ZIP 1.37 MB) [file 13428_2022_1998_MOESM1_ESM.zip › SupplementaryMaterial/Entropy_1000_K2.pdf]

Low error conditions

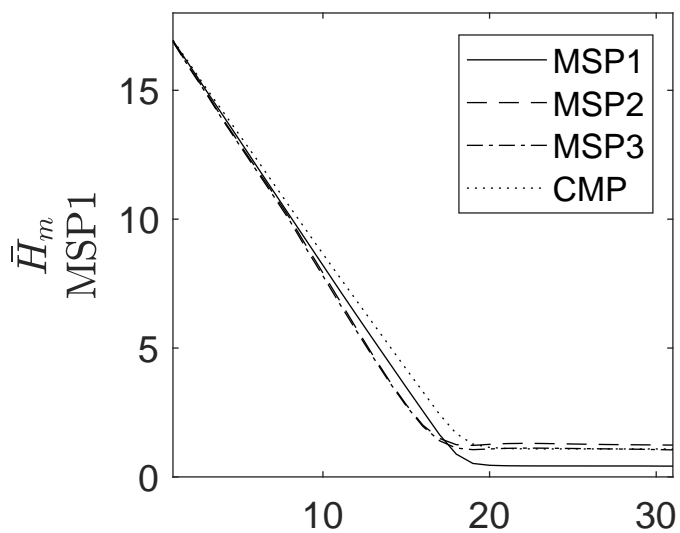

High error conditions

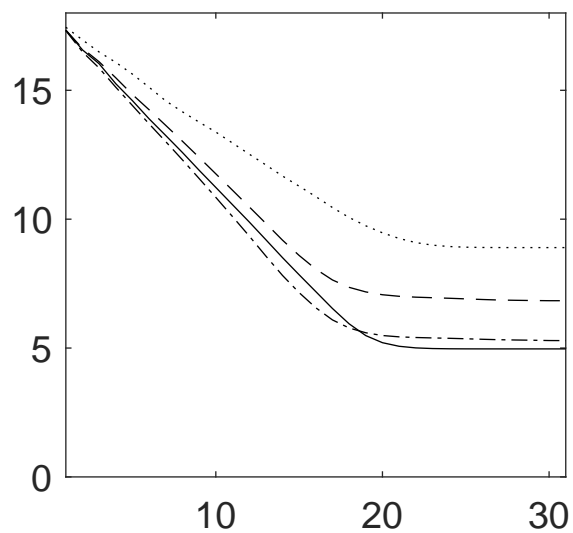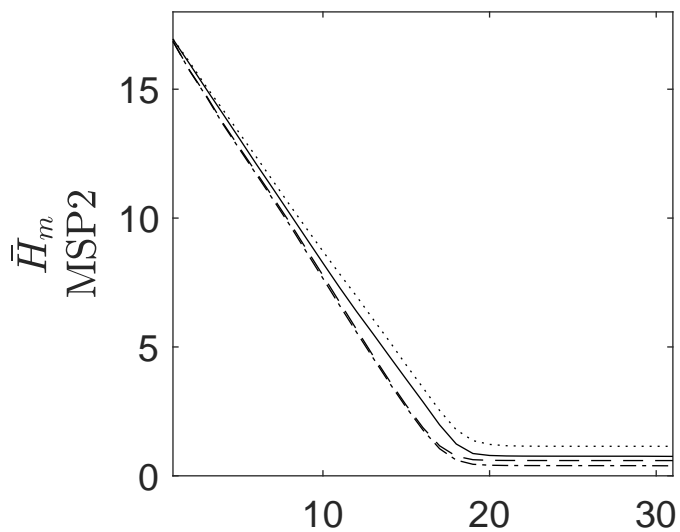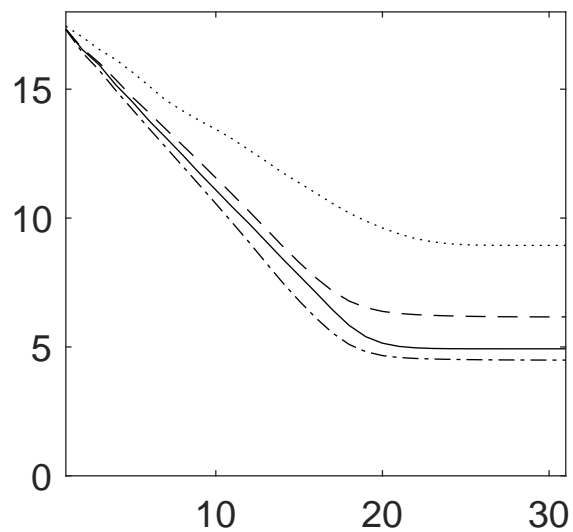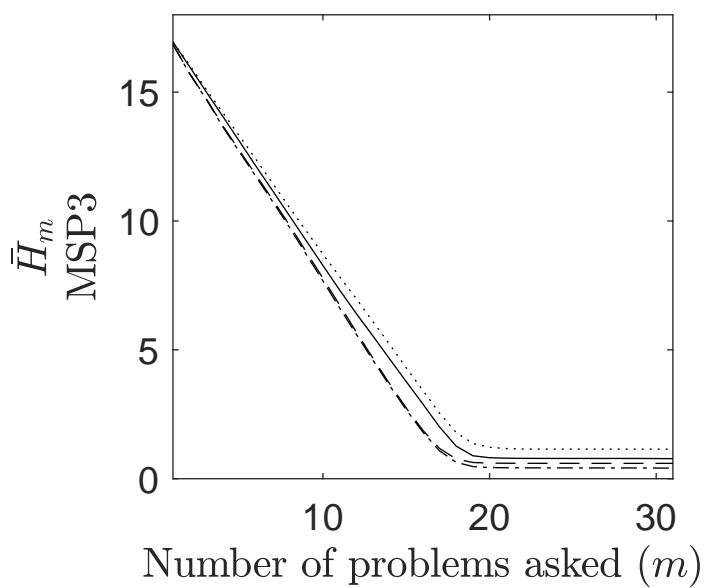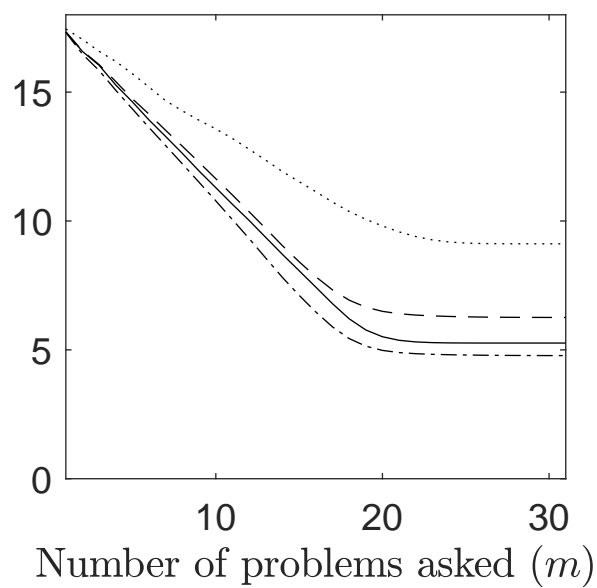

Supplement: Supplementary file 1 — (ZIP 1.37 MB) [file 13428_2022_1998_MOESM1_ESM.zip › SupplementaryMaterial/Entropy_100K_K2.pdf]

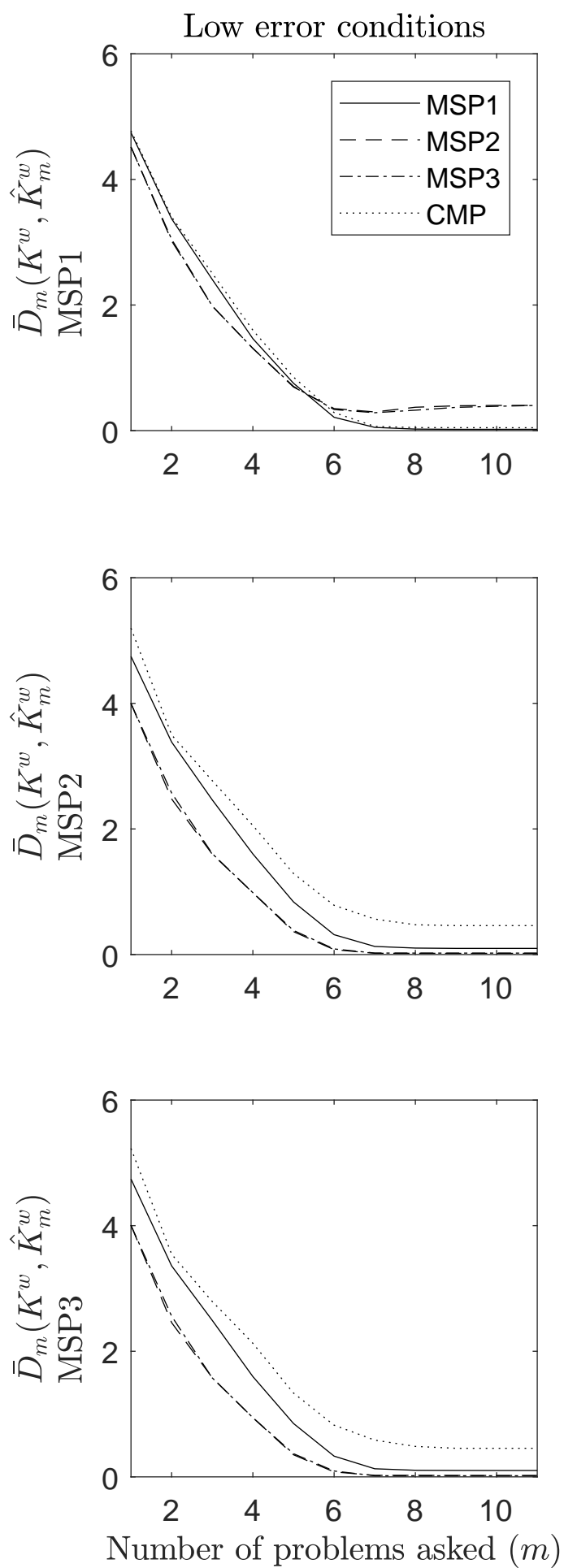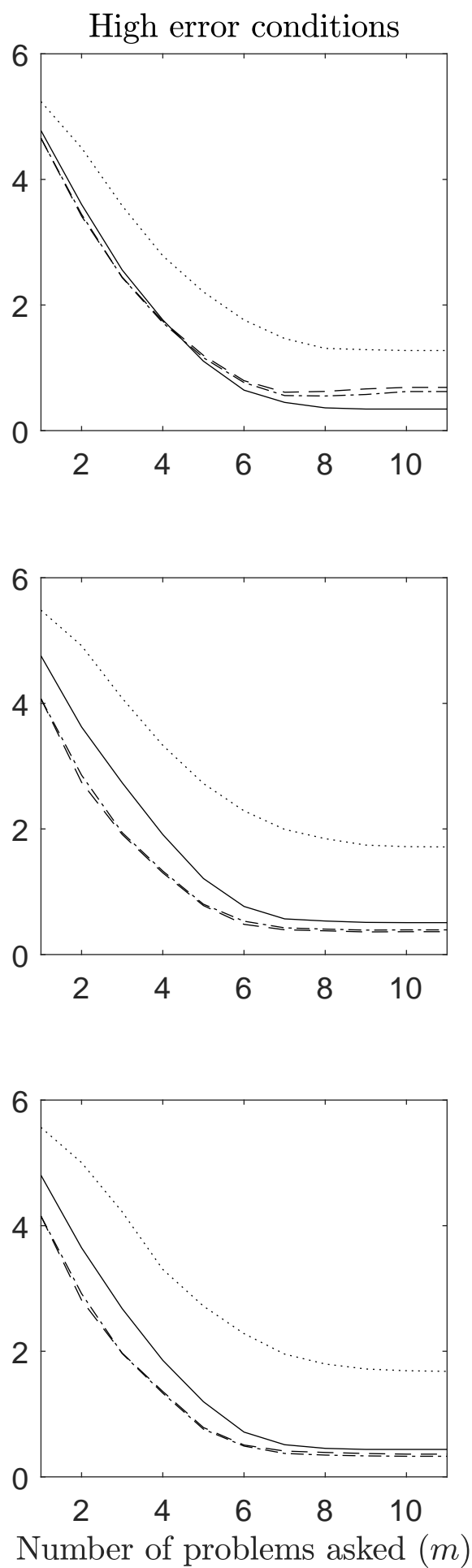

Supplement: Supplementary file 1 — (ZIP 1.37 MB) [file 13428_2022_1998_MOESM1_ESM.zip › SupplementaryMaterial/Hamming_1000_K1.pdf]

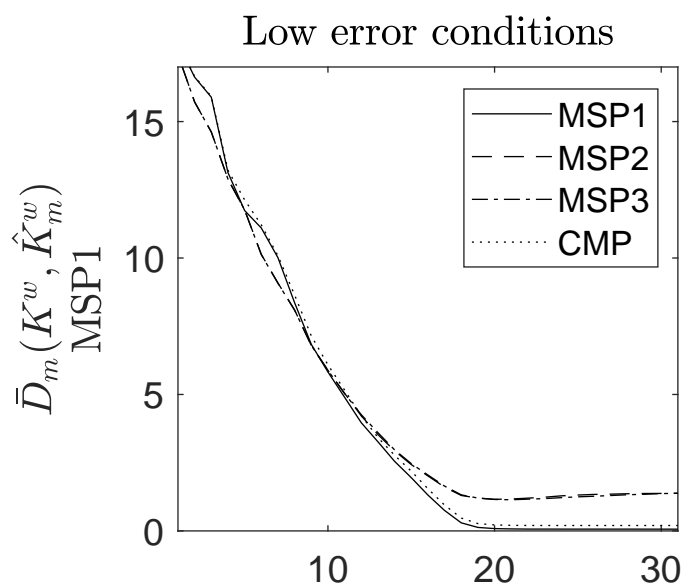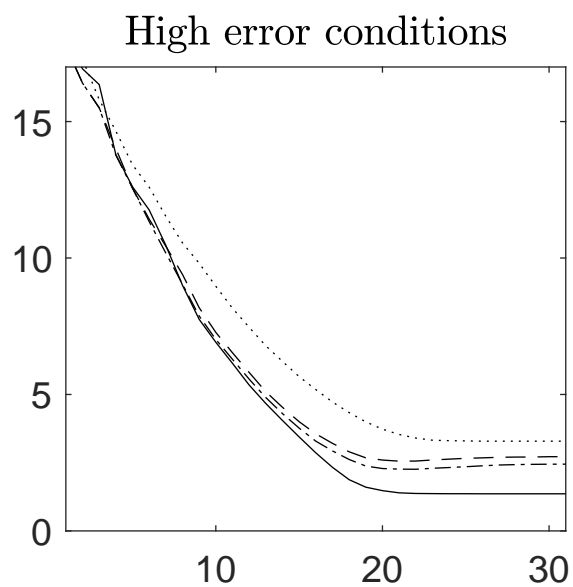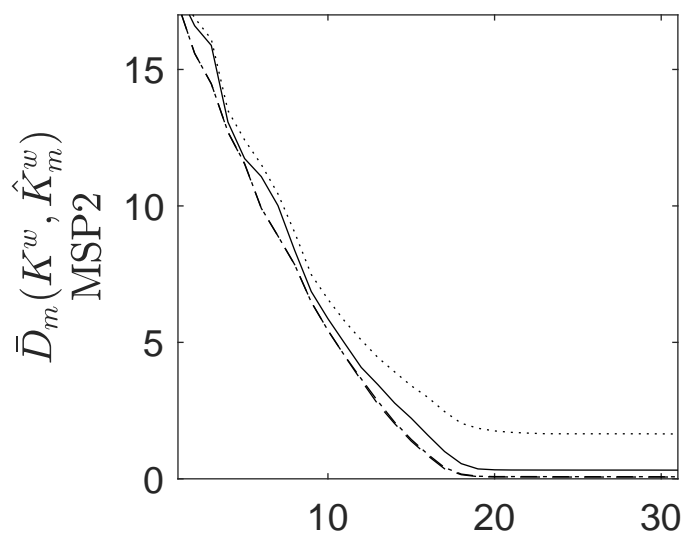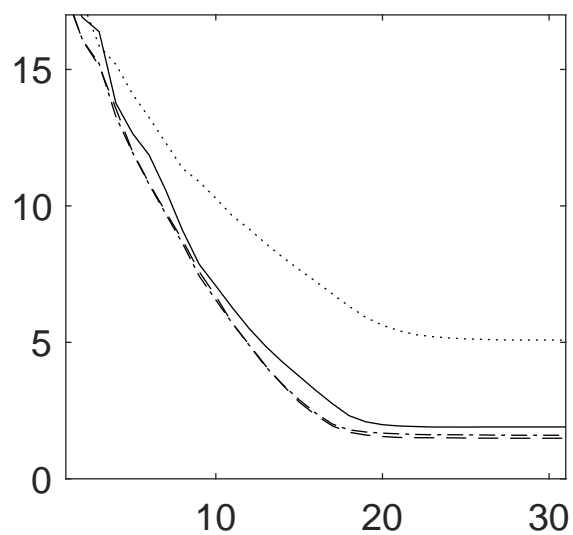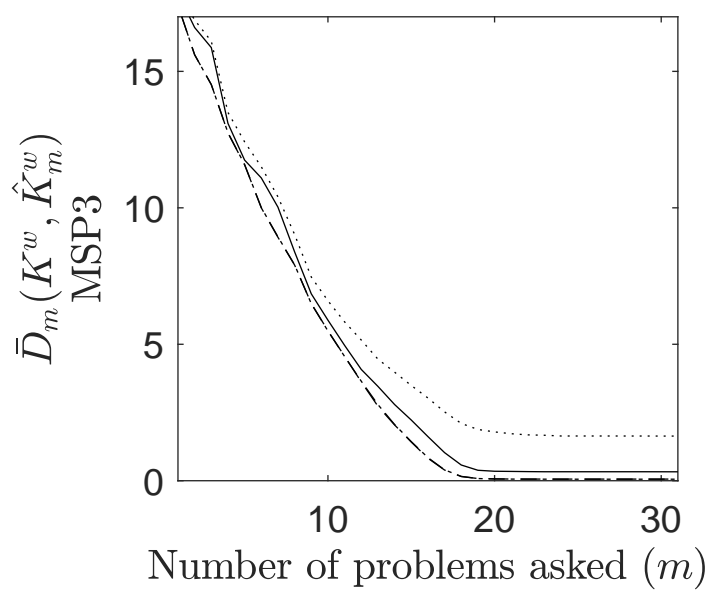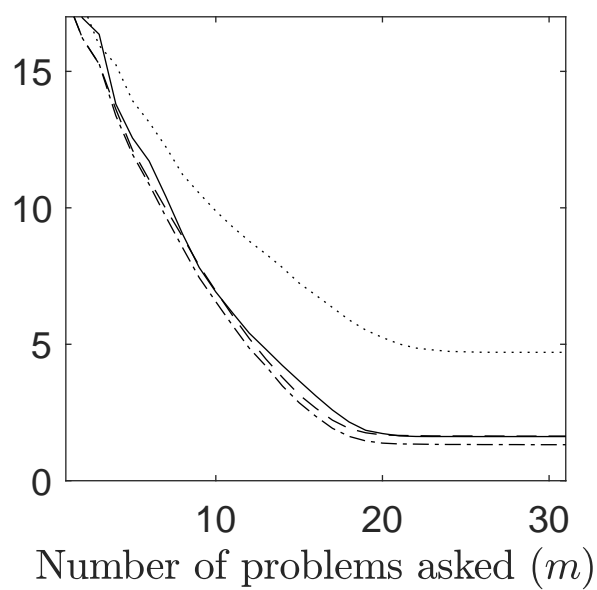

Supplement: Supplementary file 1 — (ZIP 1.37 MB) [file 13428_2022_1998_MOESM1_ESM.zip › SupplementaryMaterial/Hamming_1000_K2.pdf]

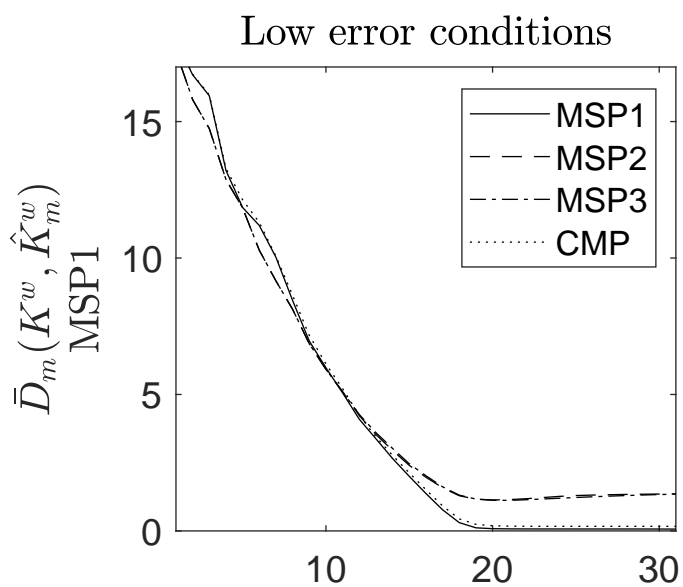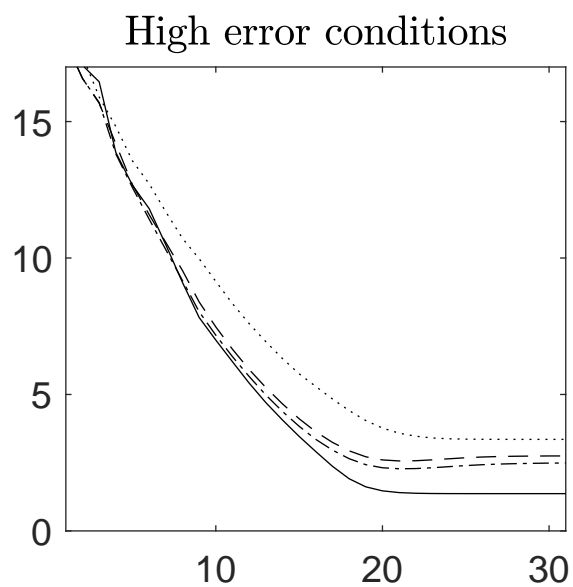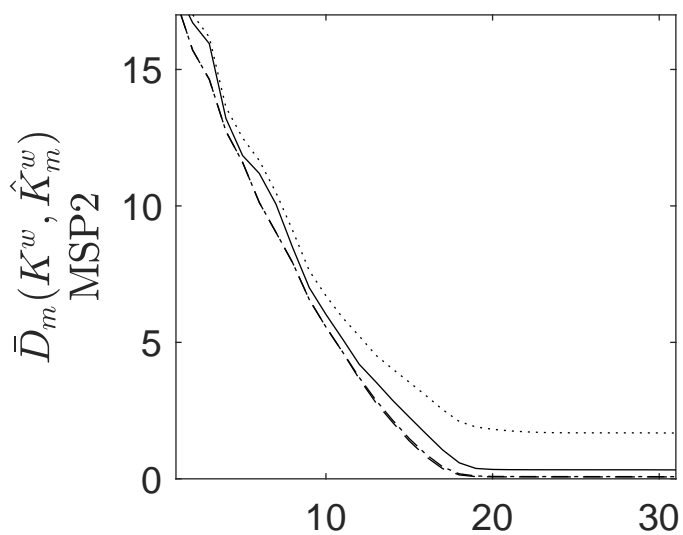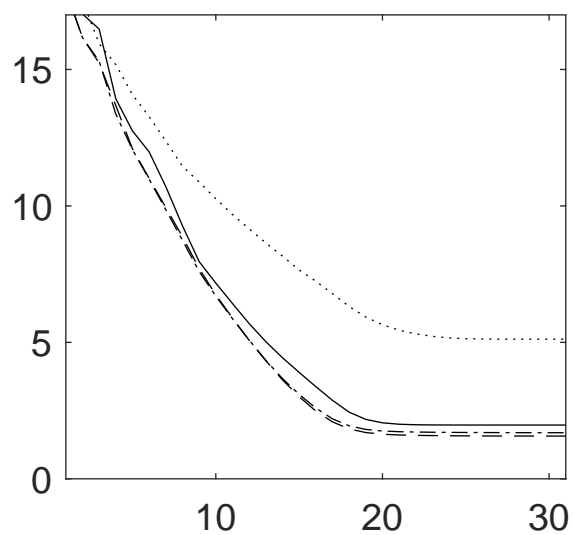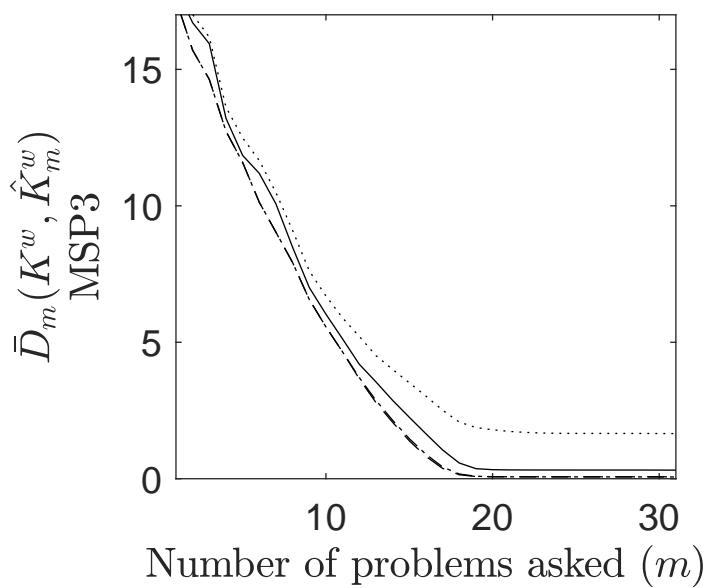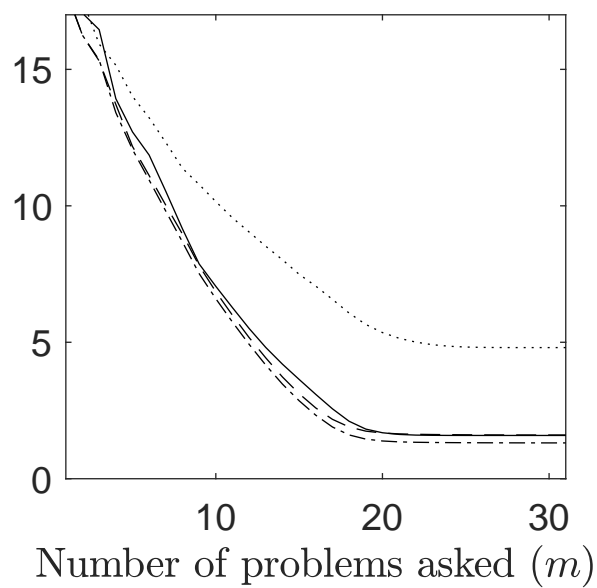

Supplement: Supplementary file 1 — (ZIP 1.37 MB) [file 13428_2022_1998_MOESM1_ESM.zip › SupplementaryMaterial/Hamming_100K_K2.pdf]
